# Supplementary material for: Variation of groundwater and mineral composition of in situ leaching uranium in Bayanwula mining area, China
Source: PLoS One. 2024 Jul 12;19(7):e0303595. doi: 10.1371/journal.pone.0303595 (PMC11244825; doi:10.1371/journal.pone.0303595)
Supplement: Attachment — Submitted filename: PONE-D-23-09655_reviewer comments.pdf [file pone.0303595.s001.pdf]

PLOS ONE

Assessment of groundwater quality with reference to minerals in situ leaching uranium in Bayanwula mining area, China

--Manuscript Draft--

**Manuscript Number:**	PONE-D-23-09655
**Article Type:**	Research Article
**Full Title:**	Assessment of groundwater quality with reference to minerals in situ leaching uranium in Bayanwula mining area, China
**Short Title:**	situ leaching uranium in Bayanwula mining area, China
**Corresponding Author:**	Malik Muhammad Akhtar, PhD, Postdoc<br>Balochistan University of Information Technology Engineering and Management Sciences<br>Quetta, PAKISTAN
**Keywords:**	In-situ leaching of uranium; Simulation of reactive transport; Water-rock interaction; Groundwater; Bayanwula mines
**Abstract:**	In the study of reactive transport during in-situ leaching of uranium, due to the complexity of hydrogeochemical reactions, mineral phases are usually simplified, and the mineral components which had been considered in the model are relatively limited. At the same time, partially penetrating well are often treated as penetrating wells, so there are some aspects to be improved for the comprehensiveness of reactive transport simulation. In this paper, a variety of minerals are added to simulate the process of in-situ leaching of uranium. The changes of groundwater composition, mineral composition and aquifer structure near the simulation area are obtained during the simulation of in-situ leaching of uranium, and the influence of the target species is given. The partially penetrating wells are discrete by grid layering. The simulation area includes two pumping-injection units extending outward and longitudinally to 300m from the mining area. A three-dimensional reactive solute transport simulation has been built using TOUGHREACT. Polygonal mesh generation is used to optimize the description of well structure. After the simulation, the observational data of three observation holes close to the mining area were selected as the fitting points. Then, the 6838-1 observation point nearest to the mining area was sampled and observed in the model, and the change of mineral component volume fraction with time at the observation point was analyzed. On this basis, the plane contour map of the mine after one year of mining is selected for observation to obtain the plane migration range of various substances. The fitting results show that the concentration correlation coefficients of the three observation holes between 0.8456~0.9984.It is concluded that calcite is the preferred mineral to be dissolved and is also one of the sources of various calcium-containing minerals, especially anhydrite. Before the dissolution of calcite, anhydrite is often dissolved from calcite. After about 0.1 years, the calcite at the observation point is dissolved, so that the in-situ leaching solution that migrates and diffuses here cannot be neutralized quickly, Therefore, the pH at observation point decreases rapidly. In this process, the main dissolved minerals in the mining process are feldspar, calcite, pyrite, Na-smectite, Ca-smectite, the main precipitation minerals are gypsum, hematite, siderite, iron dolomite and kaolinite, and the dissolved and precipitated minerals are uraninite, illite and chlorite. The range of solute migration is obtained through simulation. During the mining process, the high concentration range of K+ , Na+, Ca2+, Mg2+ and SO42- will appear at the farthest distance to 150m outside the mining area, while uranium and H+ are trapped inside the mining area, where SO42- has a long migration distance and a high concentration. The hydraulic trap effect of the pumping and injection unit will limit the migration of uranium elements in the groundwater. For H+ and SO42-, the impact on the groundwater environment will be more significant due to the high concentration in the injection fluid and the large migration range.
**Order of Authors:**	Li Haibo
	Wang Quanrong
	Malik Muhammad Akhtar, PhD, Postdoc

Powered by Editorial Manager® and ProduXion Manager® from Aries Systems Corporation

	Tang Zhonghua
**Additional Information:**	
**Question**	**Response**
**Financial Disclosure**<br><br>Enter a financial disclosure statement that describes the sources of funding for the work included in this submission. Review the submission guidelines for detailed requirements. View published research articles from *PLOS ONE* for specific examples.<br><br>This statement is required for submission and **will appear in the published article** if the submission is accepted. Please make sure it is accurate.<br><br>**Unfunded studies**<br>Enter: *The author(s) received no specific funding for this work.*<br><br>**Funded studies**<br>Enter a statement with the following details:<br>• Initials of the authors who received each award<br>• Grant numbers awarded to each author<br>• The full name of each funder<br>• URL of each funder website<br>• Did the sponsors or funders play any role in the study design, data collection and analysis, decision to publish, or preparation of the manuscript?<br>• **NO** - Include this sentence at the end of your statement: *The funders had no role in study design, data collection and analysis, decision to publish, or preparation of the manuscript.*<br>• **YES** - Specify the role(s) played.<br><br>* typeset	No Funder, Fee will be paid by authors
**Competing Interests**<br><br>Use the instructions below to enter a competing interest statement for this submission. On behalf of all authors, disclose any competing interests that could be perceived to bias this work—acknowledging all financial support and any other relevant financial or non-financial competing interests.	Authors with competing interests

This statement is required for submission and **will appear in the published article** if the submission is accepted. Please make sure it is accurate and that any funding sources listed in your Funding Information later in the submission form are also declared in your Financial Disclosure statement.<br><br>View published research articles from *PLOS ONE* for specific examples.<br><br>**NO authors have competing interests**<br><br>Enter: *The authors have declared that no competing interests exist.*<br><br>**Authors with competing interests**<br><br>Enter competing interest details beginning with this statement:<br><br>*I have read the journal's policy and the authors of this manuscript have the following competing interests: [insert competing interests here]*<br><br>* typeset	
**Ethics Statement**<br><br>Enter an ethics statement for this submission. This statement is required if the study involved:<br><br>• Human participants<br>• Human specimens or tissue<br>• Vertebrate animals or cephalopods<br>• Vertebrate embryos or tissues<br>• Field research<br><br>Write "N/A" if the submission does not require an ethics statement.<br><br>General guidance is provided below. Consult the submission guidelines for detailed instructions. **Make sure that all**	Not applicable

information entered here is included in the Methods section of the manuscript.<br><br>**Format for specific study types**<br><br>**Human Subject Research (involving human participants and/or tissue)**<br>• Give the name of the institutional review board or ethics committee that approved the study<br>• Include the approval number and/or a statement indicating approval of this research<br>• Indicate the form of consent obtained (written/oral) or the reason that consent was not obtained (e.g. the data were analyzed anonymously)<br><br>**Animal Research (involving vertebrate animals, embryos or tissues)**<br>• Provide the name of the Institutional Animal Care and Use Committee (IACUC) or other relevant ethics board that reviewed the study protocol, and indicate whether they approved this research or granted a formal waiver of ethical approval<br>• Include an approval number if one was obtained<br>• If the study involved *non-human primates*, add *additional details* about animal welfare and steps taken to ameliorate suffering<br>• If anesthesia, euthanasia, or any kind of animal sacrifice is part of the study, include briefly which substances and/or methods were applied<br><br>**Field Research**<br><br>Include the following details if this study involves the collection of plant, animal, or other materials from a natural setting:<br>• Field permit number<br>• Name of the institution or relevant body that granted permission	
**Data Availability**<br><br>Authors are required to make all data underlying the findings described fully available, without restriction, and from the time of publication. PLOS allows rare exceptions to address legal and ethical	Yes - all data are fully available without restriction

concerns. See the PLOS Data Policy and FAQ for detailed information.<br><br>A Data Availability Statement describing where the data can be found is required at submission. Your answers to this question constitute the Data Availability Statement and **will be published in the article**, if accepted.<br><br>**Important:** Stating 'data available on request from the author' is not sufficient. If your data are only available upon request, select 'No' for the first question and explain your exceptional situation in the text box.<br><br>Do the authors confirm that all data underlying the findings described in their manuscript are fully available without restriction?	
**Describe where the data may be found in full sentences. If you are copying our sample text, replace any instances of XXX with the appropriate details.**<br><br>• If the data are **held or will be held in a public repository**, include URLs, accession numbers or DOIs. If this information will only be available after acceptance, indicate this by ticking the box below. For example: *All XXX files are available from the XXX database (accession number(s) XXX, XXX.*).<br>• If the data are all contained **within the manuscript and/or Supporting Information files**, enter the following: *All relevant data are within the manuscript and its Supporting Information files.*<br>• If neither of these applies but you are able to provide **details of access elsewhere**, with or without limitations, please do so. For example:<br><br>*Data cannot be shared publicly because of [XXX]. Data are available from the XXX Institutional Data Access / Ethics Committee (contact via XXX) for researchers who meet the criteria for access to confidential data.*	Data available on request

The data underlying the results presented in the study are available from (include the name of the third party and contact information or URL).<br>• This text is appropriate if the data are owned by a third party and authors do not have permission to share the data.<br><br>* typeset	
Additional data availability information:	Tick here if your circumstances are not covered by the questions above and you need the journal's help to make your data available.; Tick here if the URLs/accession numbers/DOIs will be available only after acceptance of the manuscript for publication so that we can ensure their inclusion before publication.

Manuscript Click here to access/download;Manuscript;0 Final Paper.docx

Assessment of groundwater quality with reference to minerals in situ leaching uranium in Bayanwula mining area, China

Haibo Li[1]; Quanrong Wang[1]; Akhtar Malik Muhammad *[2]; Zhonghua Tang [1]

[1] School of Environmental Studies, China University of Geosciences, Wuhan 388 Lumo Lu, Wuhan 430074, Hubei Province, China PRC
[2] Department of Environmental Science, Faculty of Life Sciences and Informatics, Balochistan University of Information Technology, Engineering and Management Sciences, Quetta, Pakistan,

*Correspondence to: Akhtar Malik Muhammad Email: drmalikma21@gmail.com

Abstract

In the study of reactive transport during in-situ leaching of uranium, due to the complexity of hydrogeochemical reactions, mineral phases are usually simplified, and the mineral components which had been considered in the model are relatively limited. At the same time, partially penetrating well are often treated as penetrating wells, so there are some aspects to be improved for the comprehensiveness of reactive transport simulation. In this paper, a variety of minerals are added to simulate the process of in-situ leaching of uranium. The changes of groundwater composition, mineral composition and aquifer structure near the simulation area are obtained during the simulation of in-situ leaching of uranium, and the influence of the target species is given. The partially penetrating wells are discrete by grid layering. The simulation area includes two pumping-injection units extending outward and longitudinally to 300m from the mining area. A three-dimensional reactive solute transport simulation has been built using TOUGHREACT. Polygonal mesh generation is used to optimize the description of well structure. After the simulation, the observational data of three observation holes close to the mining area were selected as the fitting points. Then, the 6838-1 observation point nearest to the mining area was sampled and observed in the model, and the change of mineral component volume fraction with time at the observation point was analyzed. On this basis, the plane contour map of the mine after one year of mining is selected for observation to obtain the plane migration range of various substances. The fitting results show that the concentration correlation coefficients of the three observation holes between 0.8456~0.9984.It is concluded that calcite is the preferred mineral to be dissolved and is also one of the sources of various calcium-containing minerals, especially anhydrite. Before the dissolution of calcite, anhydrite is often dissolved from calcite. After about 0.1 years, the calcite at the observation point is dissolved, so that the in-situ leaching solution that migrates and diffuses here cannot be neutralized quickly, Therefore, the pH at observation point decreases rapidly. In this process, the main dissolved minerals in the mining process are feldspar, calcite, pyrite, Na-smectite, Ca-smectite, the main precipitation minerals are gypsum, hematite, siderite, iron dolomite and kaolinite, and the dissolved and precipitated minerals are uraninite, illite and chlorite. The range of solute migration is obtained through simulation. During the mining process, the high concentration range of K^+ , Na^+, Ca^{2+}, Mg^{2+} and SO_4^{2-} will appear at the farthest distance to 150m outside the mining area, while uranium and H^+ are trapped inside the mining area, where SO_4^{2-} has a long migration distance and a high concentration. The hydraulic trap effect of the pumping and injection unit will limit the migration of uranium elements in the groundwater. For H^+ and SO_4^{2-}, the impact on the groundwater environment will be more significant due to the high concentration in the injection fluid and the large migration range.

Keywords： In-situ leaching of uranium; Simulation of reactive transport; Water-rock interaction; Groundwater; Bayanwula mines

1. Introduction

Over half of the world population depends on groundwater for drinking and other domestic uses [1] and important for economic development [2]. Due to natural protection system, groundwater is considered contamination free water source and under-developing/developing nations must depend for domestic water supply with quality monitoring system [3]. Uranium is a radioactive element and considers toxic for human body even in small quantity [4]. Various factors could be involved to elevate the uranium concentration in aquifers such as uranium mining, groundwater depletion, excessive use of phosphate fertilizers, etc[5].In-situ leaching is an important method for uranium mining. In-situ leaching of uranium is a typical solute transport problem [6]. The whole process is under natural burial conditions, the leaching solution is injected into the ore bed through a liquid injection well, and the leaching solution contacts with the useful components in the ore to produce a chemical reaction. The resulting soluble compounds leave the chemical reaction zone under the action of diffusion and convection, migrate to the extraction pipe through infiltration, lift to the surface, process, and extract again, and finally obtain qualified products. Due to the complex chemical composition in the leaching solution of uranium in situ leaching project, the groundwater will be polluted if it is not properly controlled. The process of uranium migration involves complex hydrogeochemical processes such as oxidation-reduction, solution-precipitation, complex-dissociation, adsorption-desorption, etc[7]. So, it presents a "rolling" migration of solution-migration-sedimentation-resolution. The complex groundwater dynamic field and hydrogeochemical process control the speed, intensity, and state of solute transport, while the solute transport and its concentration change reveal the hydrodynamic characteristics and hydrogeochemical process of the in-situ leaching system [8][9]. Many researchers have studied the transport characteristics of solute and the change of components in the process of in-situ leaching, which is of great significance to understand and master the transport law of solute in the process of in-situ leaching, the acidification and oxidation process of the ore bed and the water-rock interaction that occurs, and have targeted adjustment and optimization of process parameters to achieve the purpose of improving production efficiency [10][11]. Ilton[12] explored that the form of pentavalent U in the environment was unstable, and it was the intermediate product of tetravalent uranium and hexavalent uranium, and the detectable content was often low. Uranium tetravalent may exist significantly in acidic environment. Wan [13] and Ginder-Vogel [14] reported that the existence of tetravalent uranium and hexavalent uranium as redox electric pairs was related to their hydrochemical conditions. Some other related factors may include pH, pCO2, Mg or Ca concentration. Hexavalent uranium is more inclined to oxidation environment, and tetravalent uranium is more inclined to reduction environment. A researcher [15] used MINTEQ to calculate the species of uranium-containing solution containing sulfuric acid and without sulfuric acid and compared the possible solution components in the solution system under the two conditions. Kate reported the effect of biogeochemistry on the oxidation and reduction of uranium [16]. Many researchers have studied the adsorption behavior of hexavalent uranium. Their research shows that hexavalent uranium is usually not easy to adsorb in a lower pH environment, while in

the range of 4 to 6, the adsorption behavior of hexavalent uranium will be strengthened with the increase of pH [17][18][19][20]. Steven B. Yabusaki focused on the adsorption behavior of uranium and believed that the adsorption constant was related to the concentration of uranium and the alkalinity of groundwater [21]. With the increase of uranium concentration and alkalinity, the adsorption constantly decreased. Domestic research in this area has also carried out a lot of work. Sun and other researchers conducted a precipitation experiment based on the composition of the acid in-situ leaching solution of a uranium mine in Inner Mongolia, configured a uranium-containing solution with a single impurity metal ion, and studied the precipitation behavior of uranium and impurity metal ions [22][23][24][25]. This study concluded the precipitates of Al $(OH)_3$ and Fe $(OH)_3$ accelerating the precipitation of uranium, and the precipitation of uranium in the leaching liquid was greatly affected by Al. Zheng and Chen established a hydrodynamic model for in-situ leaching of uranium in the study area with VisualModflow, and studied the effects of different pumping and injection fluid flow rates and pumping hole distances on the percolation characteristics of the solution during in-situ leaching [26][27]. Different researchers used the PHT3D software to analyze the effect of different adsorption models on the behavior of hexavalent uranium in groundwater by numerical simulation [28][29]. Chen used PHT3D to carry out numerical simulation research on the influence of calcite and pyrite minerals in pitch-bearing uranium mining areas [30].
This research work and in-situ leaching uranium simulation work have the following similarities and differences: (1) Most of them involve hydrogeochemical reactions, which provide relatively rich method support and database support for the migration simulation of in-situ leaching uranium ore. However, there are problems of inconsistent chemical reaction types and insufficient consideration of mineral types, which need to be supplemented or adjusted. (2) It is not enough to consider the ore bed structure and partially penetrating well structure in the model needs to be improved. These are the particularities of in situ leaching of uranium, so it is necessary to synthesize the existing studies and characterize the particularities of in situ leaching of uranium based on these studies.
In this study, the Bayanwula mining area is used to simulate the reactive transport of mining area, fully adding a variety of mineral components, and considering the water-rock interaction between these minerals and groundwater. In addition, the detailed mapping of the hydrogeological chemical reaction process of in-situ leaching of uranium is developed to understand the mineral transformation law in the process of in-situ leaching of uranium and obtaining the impact of in-situ leaching of uranium on the formation structure in the study area.

2. Study Area

The Bayanwula mining area is in the northeast of Inner Mongolia Autonomous Region and the northwest of Sonid Zuoqi, with a small overall area of about 90km^2. The nearest distance between the research area and the government of Sonid Zuoqi is about 30km, and well-developed transport systems are available such as railways, national roads, provincial roads, and asphalt roads. There are even airports in Erlianhot and Xilinhot

can be used to get access to the study area. Figure.1 shows the specific location of the proposed site with other informations.

(Figure 1)

2.1 Hydrogeological conditions of the test area

The exposed strata of the mine and nearby surface are the Paleogene Irdimanha group, which is largely covered by the Quaternary (Q_4) system, and the Saihan group has no outcrop. According to the borehole lithology data, the strata exposed in the mine from bottom to top include the lower section of the Saihan group (K_1s^1), the upper section of the Saihan group (K_1s^2), and the Paleogene Irdimanha group (E_2y) (Figure. 2).

(Figure 2)

The clastic rock water-bearing rock subgroup (E_2y) of the Irdimanha Formation was distributed throughout the test area and covered by the Quaternary system gradually thickness in the south and west with value 40~60m while thickness in the northeast is 60~99m. The study area contained a variety of sedimentary sandstone, sandy conglomerate, argillaceous sandy conglomerate, sandy mudstone, mudstone, etc.

The water-bearing rock subgroup (K_1s) of the clastic rock of the Saihan Formation distributed in the area and buried under the Irdimanha Formation while consists of the upper section of the Saihan Formation (K_1s^2) and the lower section of the Saihan Formation (K_1s^1) contained pore confined water. The upper section of Saihan Formation was mainly composed of sandstone, sandy conglomerate mixed with mudstone and silty mudstone mined by braided river with various thickness of 40-100m, 33.7m and 107.0m. Among the sandstone and sandy conglomerate in the middle and lower parts were loose in structure, with good connectivity, gentle occurrence, stable and continuous distribution, good water yield and permeability with a total thickness of 30~90m forming an ore aquifer. However, the water level buried depth of 13~40m, and water inflow into the well greater than $100m^3/d$. The mudstone thick at top was observed 5~20 m and distributed forming a stable water-resisting roof of the ore aquifer. The lower section of Saihan Formation is composed of mudstone and silty mudstone mixed with lignite mined in the lake and marsh. The thickness of borehole exposure was generally 5m~60m and stable water-resisting floor of the aquifer. Figure.3 shows typical hydrogeological section of the study area. The main aquifer is located at K1 stratum, and the aquifer is layered. The stratigraphic structure was relatively accurate, and the water head was very gentle, which was taken as 941m through actual measurement.

(Figure 3)

3. Model establishment and boundary condition setting

The initial conditions and boundary conditions of the model apply the initial pressure setting of the water pressure was combined with the hydrogeological conditions of the mining area. The values were computed according to the water head. Due to the low hydraulic gradient of the mining area, groundwater movement was slow. The water head distribution was relatively flat. The initial water head at the top of the aquifer was

almost 941m. The boundary conditions were adjusted based on the field visit. It was observed that the water head 300m away from the mining area was not affected by mining. Therefore, the observation points 300m away from the mining site were selected as the boundary of the model. Polygonal grids were used in the plane while rectangular grids are selected in the vertical for grid division, which were more conducive to describing the transport of reactive solute near the wellbore. The model's detailed description is given below.

3.1 Conceptual model

3.1.1 Simulation area and boundary

The simulation area was done at the end point of the mining area, that is, from the two parallel pumping and injection units at the most end point of the selected area. The pumping and injection well have distance of almost 300m and installed five observation wells in the simulation area. The division and selection of the simulation area were mainly based on the following:

(1) Based on the distribution characteristics of the flow field around the mining area. The distribution of pumping and injection wells was very tidy. So, the inflow and outflow must be same from the injection and pumping well. Moreover, the actual investigation and numerical simulation study of similar mining models showed that the flow field formed by such mining areas is characterized by parallel flow gradually outward from the mining area boundary, and the water level lines are approximately parallel to each other and gradually increasing. Therefore, the two long lateral boundaries on both sides of the simulation area were treated as impermeable boundaries.

(2) The groundwater flow in the study area was very small, and the change of local flow field is mainly controlled by the mining wells.

(3) Each production area was pumped in the mode of four injections wells and one pumping well. The flow field developed by the pumping well was the convergence of four injection wells to one pumping well, which was like the four-leaf petal-shaped distribution. In the direction of the connecting line of the injection wells, it could be approximated as the water barrier boundary.

(4) There was an observation well located 300m away from the mining area. According to the value of the water table at the observation well, the fluctuation in water table was slightly affected due to activities at the mining area. Therefore, at the distance of 300m, it could be regarded as a fixed water head boundary to control the pressure boundary.

Based on the above analysis, the boundary of the simulation area was categorized into three impermeable boundaries and one fixed head boundary shown in Figure.4. The orange area is a mining area and has two pumping and one injection wells while the white area is the periphery of the mining area, without ore. The red point in the figure represents the injection well, and the blue dot represents the pumping well.

(Figure 4)

3.1.2 Well flow rate

Accordingly, the flow rate of the injection well diverged to the inside of the production

area. If the pumping fluid was arranged inside the study area, the injection well just took the flow rate of a single well but was in the edge area. According to the actual situation, the injection well had the problem of internal and external flow distribution. Combined with the distribution characteristics of the flow field of the extraction well, if the injection well was at the edge of the boundary, half of the flow rate of a single well was taken while if the injection well was at the corner of the boundary than one quarter of the flow rate was taken. The pumping well was in the center of the pumping well and the flow rate of a single well could be taken. According to the data of the mining area, the flow rate of the injection well of the simulated area was obtained through calculation of various parameters.

Table 1. Well flow rate.

Injection well flow rate	Pumping well flow rate
176.486 m^3/d	264.720 m^3/d

3.1.3 Parameter partition

Parameter zoning mainly included permeability zoning and mineral composition zoning. Due to the little change of initial permeability within the region in this simulation, the initial permeability in the simulation zone remains the same, while mineral composition was divided into uranium and non-uranium zones. According to the mining area scope in the model generalization (Figure 4), the ore and ore-free zones were represented with different colors. With reference to the regional geological report, the spatial location partition and the initial parameter setting of the model was verified to the mineral component information.

3.2 Mathematical model

In this study, TOUGHREACT software was used to develop models and simulate. TOUGHREACT [31][32] was developed by introducing reactive geochemistry into the framework of TOUGH2V2[33]. TOUGHREACT has been applied to simulate many questions of subsurface hydrological and biogeochemical environments [34][35]. The migration of reactive solute was the coupling of convective dispersion and hydrogeochemical reaction during subsurface flow system process. Based on the conservation of mass and energy and Darcy's law, a mathematical model of groundwater flow was established. In the TOUGH series of software, mass conservation equations of material components (groundwater, salt, and gas components) were adopted. Through this method, a multiphase system was well described, and the transformation of the same substance between different phase states was elaborated. In this process, it was necessary to consider the thermodynamic properties of the phase states and components. The governing equation used in the model is as follows:

The equation is established from the conservation of mass:

$$\frac{\partial M_\kappa}{\partial t} = -\nabla F_\kappa + q_\kappa$$

For water:

$$F_w = x_{wl}\rho_l\overrightarrow{u_l} + x_{wg}\rho_g\overrightarrow{u_g},\ q_w = q_{wl} + q_{wg}$$

For air:

$$F_c = x_{cl}\rho_l\overrightarrow{u_l} + x_{cg}\rho_g\overrightarrow{u_g},\ q_c = q_{cl} + q_{cg} + q_{cr}$$

For heat:

$$M_h = \varphi\left(S_l\rho_l U_l + S_g\rho_g U_g\right) + (1-\varphi)\rho_s U_s$$

$$F_h = \sum_{\beta=l,g} h_\beta\rho_\beta\overrightarrow{u_\beta} - \lambda\nabla T,\ q_h$$

According to Darcy's law:

$$\overrightarrow{u_\beta} = -k\frac{k_{r\beta}}{\mu_\beta}\left(\nabla P_\beta - \rho_\beta\vec{g}\right)$$

For chemical components in the liquid phase:

$$M_j = \varphi S_l c_{jl}, \tau = \varphi^{1/3}S_l^{7/3}$$

$$F_j = c_{jl}\overrightarrow{u_l} - \tau\Phi s_l D_l\nabla c_{jl},\ q_j = q_{jl} + q_{jg} + q_{jr}$$

The main chemical reaction mathematical models in the simulation include equilibrium mineral and kinetic mineral. Equilibrium mineral model is:

$$\Omega_m = K_m^{-1}\prod_{j=1}^{N_c} c_j^{\nu_{mj}}\gamma_j^{\nu_{mj}}$$

Kinetic mineral model is:

$$r_n = \pm k_n A_n\left|1 - \Omega_n^\theta\right|^\eta$$

$$k_n = k_{25}^{nu}exp\left[-\frac{E_a^{nu}}{R}\left(\frac{1}{T} - \frac{1}{298.15}\right)\right] + \sum_i\left\{k_{25}^i exp\left[-\frac{E_a^i}{R}\left(\frac{1}{T} - \frac{1}{298.15}\right)\right]\right\}\prod_j a_{ij}^{n_{ij}}$$

3.3 Mesh generation

The distance between the injection wells was maintained almost 50m, and the pumping wells were in the middle of the study area and four injection wells were installed in its surrounding. There were six injection wells and two pumping wells installed to conduct this study. To better describe the underground water flow field and chemical field near the well, polygon grid was used for plane section and rectangular grid was used for vertical section which is divided into six layers. The fifth layer covered the mining area which represents the ore layer. The division is shown in Figure.5 and Figure.6. The actual size of the grids in the east-west, north-south and vertical length is 300m, 100m and 60m, respectively. The vertical grid division information is shown in Table.2. The red part of the 3D grid is the ore bed (Figure.6).

Table 2. Aquifer stratification information

Aquifer number	1	2	3	4	5	6
Top (m)	60	40	28.5	18.5	8.5	2.5
Bottom (m)	40	28.5	18.5	8.5	2.5	0
Thickness (m)	20	11.5	10	10	6	2.5

(Figure 5)

(Figure 6)

3.4 Initial parameters of the model

3.4.1 Initial temperature and pressure

The initial temperature of the aquifer was observed 9℃ during the sampling collection. The lithology formation of the simulation is in the shallow layer, and the regional geological survey and the research area had no strong geothermal gradient. And the temperature change of the formation is small, the effect was not very good. So, this study did not consider the impact of temperature changes on the system. The whole process was an isothermal simulation.

3.4.2 Initial water and boundary water

3.4.2.1 Initial water

To determine the initial water in the simulation process, some water samples were collected in the mining area. The distributed samples were collected from upstream and far from the mining area. The initial water composition was observed include in table.3.

Table 3. The concentration of various elements in Initial water

Index	Concentration
Na（mg/L）	495
K（mg/L）	8.46
Ca（mg/L）	76.6
Mg（mg/L）	55.2
SO_4^{2-}（mg/L）	407
CI^-（mg/L）	410
HCO_3^-（mg/L）	1110
Fe（mg/L）	0.747
U（μg/L）	30.1

3.4.2.2 Boundary water

To determine the chemical composition of boundary water in the simulation process, samples were taken from the area where the mining area is located, and the samples were injected from the interior of the mining area. According to the composition of injection liquid, the concentration of different elements of boundary water is shown in table.4.

Table 4. The concentration of various elements in boundary water

Index	Concentration
Na（mg/L）	1950
K（mg/L）	533

Ca（mg/L）	441
Mg（mg/L）	916
SO_4^{2-}（mg/L）	27300
Cl^-（mg/L）	464
HCO_3^-（mg/L）	35
Fe（mg/L）	1580
U（mg/L）	0.79

3.4.2.3 Initial minerals

The initial mineral composition is divided into feldspar minerals, clay minerals, uranium minerals, iron minerals and carbonate minerals. The initial mineral composition of the ore layer can be generalized as follows:

Table 5 Initial mineral composition

name	Chemical formula	Initial mineral volume fraction of ore body	Initial mineral volume fraction of surrounding rock
Quartz	SiO_2	0.48686	0.48686
K-feldspar	$KAlSi_3O_8$	0.21550	0.21550
Oligoclase	$CaNa_4Al_6Si_{14}O_{40}$	0.01134	0.01134
Na-smectite	$Ca_{0.145}Mg_{0.26}Al_{1.77}Si_{3.97}O_{10}(OH)_2$	0.08391	0.08391
Ca-smectite	$Na_{0.29}Mg_{0.26}Al_{1.77}Si_{3.97}O_{10}(OH)_2$	0.02597	0.02597
Illite	$K_{0.6}Mg_{0.25}Al_{1.8}(Al_{0.5}Si_{3.5}O_{10})(OH)_2$	0.02137	0.02137
Kaolinite	$Al_2Si_2O_5(OH)_2$	0.01520	0.01520
Uraninite	UO_2	0.01143	0.00000
Hematite	Fe_2O_3	0.01880	0.01880
Pyrite	FeS_2	0.00245	0.00028
Calcite	$CaCO_3$	0.03320	0.01140

3.4.2.4 Secondary minerals

(1) Gypsum and anhydrite

In acid in-situ leaching production, when high-acidity leaching solution is injected into the ore layer through the injection hole, sulfuric acid will react with limestone in the ore layer，produce anhydrite ($CaSO_4$) or gypsum ($CaSO_4 \cdot 2H_2O$) precipitation. The reaction equation is as follows:

$$CaCO_3 + 2H^+ = Ca^{2+} + H_2O + CO_2(g)$$

$$Ca^{2+} + SO_4^{2-} + 2H_2O = CaSO_4 \cdot 2H_2O(s)$$

(2) Hematite

Hematite is also the main mineral that produces chemical precipitation in the in-situ leaching area of Bayanwula uranium mine. When there is a certain amount of Fe^{3+} in the water, it is also easy to form hematite precipitation. The reaction formula is as follows:

$$2Fe^{3+} + 3H_2O = Fe_2O_3 + 6H^+$$

At the same time, in addition to the minerals found in the above survey, some possible minerals commonly found in the TOUGHREACT database, including muscovite, dolomite, siderite, and ankerite, have also been added.

Table 1 Secondary mineral in simulation

mineral	Chemical formula
Muscovite	$KAl_2(AlSi_3O_{10})(OH)_2$
Dolomite	$CaMg(CO_3)_2$
Siderite	$FeCO_3$
Ankerite	$CaMg_{0.3}Fe_{0.7}(CO_3)_2$

3.4.2.5 Relative permeability and capillary pressure calculation model

The calculation model of relative permeability and capillary pressure in the model adopted by Van Genuchten-Mualem model[36]. The calculation formula was used shown in the table 7.

Table 7. Relative permeability and capillary pressure of the model

Parameters	value
Aquifer thickness(m)	60
Rock grain density(kg/m3)	2600
Porosity	0.085
K: absolute permeabity(m^2)	3×10^{-12}
Temperature(℃)	9
Rock grain specific heat(J/(Kg·℃))	920
Formation heat conductivity(W/(m·℃))	2.51
Pressure(MPa)	5
Salinity(NaCl mass fraction)	0.03
Liquid relative permeability, use the Van Genuchten-Mualem model[36][37]: $k_{rl} = \sqrt{S^*}\left\{1-\left[1-[S^*]^{1/\lambda}\right]^{\lambda}\right\}^2$, $S^* = (S_l - S_{lr})/(S_{ls} - S_{lr})$	
λ:exponent	0.457
S_{lr}:residual gas saturation	0.05
S_{ls}	1
Gas relative permeability, Corey[38]: $k_{rg} = (1-\hat{S})^2(1-\hat{S}^2), \hat{S} = (S_l - S_{lr})/(1 - S_{lr} - S_{gr})$	
S_{gr}:residual gas saturation	0.05
Capillary pressure, use the Van Genuchten-Mualem model[36]: $P_{cap} = -P_0\left([S^*]^{-1/\lambda} - 1\right)^{1-\lambda}$ $S^* = (S_l - S_{lr})/(S_{ls} - S_{lr})$	
λ	0.457
S_{lr}, residual water saturation	0.3

S_{ls}	0.999
P_{max}	1×10^{7}
P_0	1.96×10^{4}

3.4.4 Thermodynamic and kinetic parameters of mineral reaction

The mineral reaction mainly selected the dissolution and precipitation of relevant minerals, and its chemical reaction parameters are as follows the table 8.

Table 8. The mineral reaction kinetic parameters used in the Model

Minerals	Chemical formula	Surface area(cm2/g)	Parameters for kinetic rate law							
			Neutral mechanism		Acid mechanism			Base mechanism		
			k_{25}(mol/ m^2/s)	E_a (KJ/mol)	k_{25}(mol /m^2/s)	E_a (KJ/mol)	N (H^+)	k_{25}(mol/ m^2/s)	E_a (KJ/mol)	n(OH^-)
Calcite	$CaCO_3$	9.8	Equilibrium							
Anhydrite	CaSO4	9.8	Equilibrium							
Quartz	SiO_2	9.8	1.023×10^{-14}	87.7						
Illite	$K_{0.6}Mg_{0.25}Al_{1.8}(Al_{0.5}Si_{3.5}O_{10})(OH)_2$	151.6	1.660×10^{-13}	35.0	1.047×10^{-11}	23.6	0.34	3.020×10^{-17}	58.9	-0.4
K-feldspar	$KAlSi_3O_8$	9.8	3.890×10^{-13}	38.0	8.710×10^{-11}	51.7	0.5	6.310×10^{-22}	94.1	-0.823
Chlorite	$Mg_{2.5}Fe_{2.5}Al_2Si_3O_{10}(OH)_8$	20.0	3.020×10^{-13}	88.0	7.762×10^{-12}	88.0	0.50			
Na-smectite	$Na_{0.29}Mg_{0.26}Al_{1.77}Si_{3.97}O_{10}(OH)_2$	151.6	1.660×10^{-13}	35.0	1.047×10^{-11}	23.6	0.34	3.020×10^{-17}	58.9	-0.4
Kaolinite	$Al_2Si_2O_5(OH)_4$	23.0	6.918×10^{-14}	22.2	4.898×10^{-12}	65.9	0.777	8.913×10^{-18}	17.9	-0.472
Ca-smectite	$Ca_{0.145}Mg_{0.26}Al_{1.77}Si_{3.97}O_{10}(OH)_2$	151.6	1.660×10^{-13}	35.0	1.047×10^{-11}	23.6	0.34	3.020×10^{-17}	58.9	-0.4
Gypsum	$CaSO_4$	9.8	1.6218e-07							
Pyrite	FeS_2	12.9	2.52×10^{-12}	62.76						
Oligoclase	$CaNa_4Al_{1.77}Si_{3.97}O_{10}(OH)_2$	10.0	1.4454e-13	69.80						
Hematite	Fe_2O_3	12.9	2.512×10^{-15}	66.2	4.0738×10^{-10}	66.2	1			
Muscovite	$KAl_2(AlSi_3O_{10})(OH)_2$	152.0								
Siderite	$FeCO_3$	10.0	1.26×10^{-9}	62.76	6.46×10^{-4}	36.1	0.5			
Dolomite	$CaMg(CO_3)_2$	12.9	2.52×10^{-12}	62.76	2.34×10^{-7}	43.54	1			
Ankerite	$CaMg_{0.3}Fe_{0.7}(CO_3)_2$	9.8	1.26×10^{-9}	62.76	6.46×10^{-4}	36.1	0.5			
Magnesite	$MgCO_3$	10.0								

3.4.5 Model simulation accuracy and verification

The fitting accuracy of the concentration field uses the linear correlation coefficient to measure the goodness of fit of the model. The expression of the linear correlation coefficient is as follows：

$$r = \frac{\sum_{i=1}^{n}(C_i - \overline{C_i})(b_i - \overline{b_i})}{\sqrt{\sum_{i=1}^{n}(C_i - \overline{C_i})^2}\sqrt{\sum_{i=1}^{n}(b_i - \overline{b_i})^2}}$$

Where, C_i is the simulated concentration, and $\overline{C_i}$ is the mean value of the simulated value; b_i is the observed concentration, $\overline{b_i}$ is the mean value of the observed value.

Near the simulation area, there are three typical observation holes: 6838-1, 6838-2, 6838-3. Three observation holes were sampled respectively, of which pH value is for field test, and SO_4^{2-}, Na^+ and K^+ are sent to the mining area laboratory for test and analysis.

Figure 7 shows the curve of each test index concentration of each observation well. From the figure, the simulated values of most points are close to the observed values, and the change in trend of the simulated values is consistent with the observed values, indicating that the model established this time reflect the actual hydrogeological conditions in the study area and can be used to predict the groundwater flow field.

(Figure 7)

As shown in Table 9, there are 54 data points assigned in the study area and 9 points for each well. The maximum error of pH of Well 6838-1 is 1.21, the minimum error is 0.123, and the average error is 0.615; The maximum error of SO_4^{2-} concentration is 0.05mol/kg, the minimum error is 0.0003mol/kg, the average error is 0.0202mol/kg, and the average relative error is 0.182. The maximum concentration error of Na^+ is 0.03mol/kg, the minimum error is 0.00205mol/kg, the average error is 0.0138mol/kg, and the average relative error is 0.171; The maximum error of K^+ concentration is 0.0445 mol/kg, the minimum error is 0.00005 mol/kg, the average error is 0.0188 mol/kg, and the average relative error is 0.227.

The maximum error of pH at Well 6838-2 is 1.2, the minimum error is 0.035 while the average error and average relative error are 0.525 and 0.146, respectively. The maximum error of SO_4^{2-} concentration is 0.04mol/kg and the minimum error is 0.0003mol/kg while values of the average error and average relative error are 0.0174mol/kg and 0.214 mol/kg. In the case of Na+ concentration the maximum error, minimum error, average error and average relative error are 0.0269mol/kg, 0.002mol/kg, 0.0148mol/kg, and 0.172 mol/kg, respectively. The maximum error of K+ content is 0.0101mol/kg and the minimum error is 0.00005 mol/kg. The average error of K+ is 0.0907mol/kg while the average relative error is 0.9202.

The maximum error and minimum error of pH of Well 6838-3 is 1.48 and 0.065, respectively. The average error and average relative error of pH are 0.498, and 0.104, respectively. The maximum error of SO_4^{2-} content is 0.025mol/kg and the minimum error is 0.0003 mol/kg while the average error and average relative error are 0.0735

mol/kg, and 0.157, respectively. The maximum error and minimum error of Na+ content is 0.045mol/kg and 0.002mol/kg, respectively. However, the average error and average relative error of Na+ content is observed 0.0177mol/kg and 0.181. The value of maximum error, minimum error, average error and average relative error of K+ content is computed 0.055mol/kg, 0.00005mol/kg, 0.0203mol/kg, and 0.420, respectively. The correlation coefficients of simulated and observed values are calculated in Table 3.9. The correlation coefficients of three observed well is between 0.8456 and 0.9984. The simulation accuracy was more than 80% in this study.

Table 9. Statistical table of fitting error of concentration field

Observed well	Observed data point	Index	Max error	Min error	Average error	Average relativity error	correlation coefficient
	9	pH	1.21	0.123	0.615	0.037	0.9984
	9	SO_4^{2-}	0.05	0.0003	0.0202	0.182	0.9116
	9	Na^+	0.03	0.00205	0.0138	0.171	0.9282
6838-1	9	K^+	0.0445	0.00005	0.0188	0.227	0.9438
	9	pH	1.2	0.035	0.525	0.146	0.9932
	9	SO_4^{2-}	0.04	0.0003	0.0174	0.214	0.9113
	9	Na^+	0.0269	0.002	0.0148	0.172	0.8798
6838-2	9	K^+	0.0101	0.00005	0.0907	0.9202	0.9202
	9	pH	1.48	0.065	0.498	0.104	0.9953
	9	SO_4^{2-}	0.025	0.0003	0.0735	0.157	0.9588
	9	Na^+	0.045	0.002	0.0177	0.181	0.8456
6838-3	9	K^+	0.055	0.00005	0.0203	0.420	0.9075

4. Results and discussions

4.1 Mineral volume changes at observed well.

Through the analysis of the simulation results of the change in the volume fraction of the mineral species at the observation point, the dissolution and precipitation state of various minerals can be clearly identified. A positive change in the volume fraction of the mineral indicates that the mineral has precipitated, and a negative change in the volume fraction of the mineral indicates that the mineral has dissolved. To observe the dissolution and precipitation of various minerals, the simulated values of the volume fraction changes of several mineral components in the observation well 6838-1 nearest to the mining area were analyzed. According to the simulation results at the location of observation well 6838-1, anhydrite is a precipitation mineral. After mining, anhydrite began to precipitate, and the precipitation rate gradually increased. At about 0.1 years, the precipitation reached the peak, and the volume fraction increased to the maximum, and then the volume integral number of anhydrites decreased. At about 0.8 years, the volume fraction of anhydrite returned to the initial value. Calcite is one of the minerals that undergo severe dissolution. After mining, the calcite began to dissolve, and the dissolution rate gradually increased. When it reached about 0.1 years, the volume fraction of calcite decreased the most. The actual simulation results show that at this

time, the calcite at the observation point had been completely dissolved. The change of the volume fraction of uraninite shows a peak shape and reaches the peak in 0.1 year. The maximum increase of the volume fraction can reach 0.008. This is because the solution brought by the outward migration of the mining fluid in the mining area contains high concentration of uranium ore solution, so uraninite re-precipitated with the increase of pH, and at the same time, uraninite showed a trend of increasing first and then decreasing, At the same time, it was noted that the precipitated uraninite began to dissolve after the consumption of calcite was completed. From this feature, the acidic solution dissolved calcite first, which slowed down the dissolution of uraninite. Hematite began to precipitate after the mining was started. This is because the concentration of hematite in the mining fluid is high. As the mining fluid moved outward, hematite gradually precipitated. However, the precipitation trend of hematite also decreased sharply in 0.1 year. This is because the calcite minerals in the observation point are consumed at this time, so the pH value at the observation point dropped sharply, and the water environment at the observation point also weakened the precipitation trend of hematite minerals. K-feldspar minerals also entered the dissolution state after 0.1 years of mining, indicating that the dissolution of calcite affected the dissolution of potassium feldspar. Gypsum means that after mining, the volume fraction had been increasing, and the precipitation trend had slowed down sharply by about 0.8 years. At the same time, compared with the precipitation trend of anhydrite minerals, anhydrite precipitation is completely dissolved again by about 0.8 years, indicating that a large part of gypsum precipitation comes from the dissolution of anhydrite. When anhydrite was completely dissolved, gypsum also stopped precipitation substantially. Oligoclase and pyrite were also affected by the dissolution of calcite, and these two minerals began to dissolve significantly at 0.1 year. In clay minerals, illite and chlorite showed a trend of precipitation before dissolution, while Ca-smectite and Na-smectite were continuously dissolved, while kaolinite was continuously precipitated. Their dissolution and precipitation changed abruptly at 0.1 year, indicating that the dissolution and precipitation of these five minerals were also related to calcite. Siderite and ankerite were precipitated first and then dissolved, which was also due to the consumption of calcite at the observation point in 0.1 year, so that the two minerals were rapidly dissolved. Muscovite mineral is like hematite, which was precipitated first and then unchanged. Through the analysis of the dissolution and precipitation state of these minerals, at the observation point, the reaction of mineral composition existed in the order, in which calcite reacted first and became the source of anhydrite and gypsum minerals, while other minerals reacted further after calcite reaction (Figure 8).

(Figure 8)

By means of numerical simulation of mining situation in the mining area, the change of the migration situation of the chemical component of groundwater and the composition of each mineral component in groundwater were obtained, and the change of the pore permeability structure was obtained, and the simulation results can be observed to analyze the trend and influence factors of them.

4.2 The extent of transport and diffusion

4.2.1 The transport and diffusion of uranium

When the acid mining fluid was injected into the aquifer, the uranium mineral was dissolved in the groundwater, and this was the major mechanism of the in-site leaching. The blue area in the picture means uranium dissolution, and the red area means uranium precipitation. The maximum increase in the volume fraction of uranium ore can exceed 0.006% but not exceeded 0.008%. The volume fraction of uranium ore can be reduced by more than 0.01%. This result shows that, from the overall perspective, uranium ore was mainly dissolved. From the simulation results, the uranium ore was mainly dissolved near the injection well, which was the main source of uranium element in the mining area and its nearby liquid phase, and the volume fraction of uranium was reduced by 0.01% near the injection hole. The volume fraction of uranium can increase to 0.006% near the pumping well. This is e with the flow of groundwater, the acid in the leaching solution was gradually consumed, which lead to the decline of pH, so the previously dissolved uranium precipitated again. In the outer area of the mining area, there was no uranium in the aquifers.

The simulation results show that after a year of mining, the concentration of pH in the solution and the increase of the uranium concentration and the increase of the uranium content were reprecipitated. From the distribution of uranium concentration, the uranium concentration was higher near the pumping well but not highest (Figure 9). The highest concentration of UO_2^{2+} appeared between the injection well and pumping well. The max concentration of UO_2^{2+} exceeded 0.004mol/kg (Figure 10).

(Figure 9)

(Figure 10)

4.2.2 The diffusion and transport of SO_4^{2-}

In the process of acid leaching, the amount of sulphuric acid is large, so the SO_4^{2-} concentration is an object that needs to be seriously described. In the area of the mining area, the amount of uranium was reduced, and the degree of volume reduction was more than 0.01%. The blue area shows the area where the mineral is dissolved, and the region of the uranium mining is dissolved, and as the solution is moved to the extraction well, the uranium mine is gradually precipitated, but the amount of precipitation is far less than that of the mine, which is far less than the degree of solubility of the liquid well, which can be more than 0.006% but not more than 0.008% (Figure 11). The simulation results show that after a year of mining, the concentration of pH in the solution and the increase of the uranium concentration and the increase of the uranium content were reprecipitated. From the distribution of uranium concentration, the uranium concentration was low, and the uranium concentration was high near the pumping well.

(Figure 11)

4.2.3 The diffusion and transport of H^+

The concentration of H^+ is an important factor affecting in situ leaching mining. After

one year of mining, the pH was changed as shown in the figure. In the interior of the mining area, the pH value was relatively low. As the distance from the mining area increased, the pH value gradually increased. It can also be seen from the figure that if the range of lower pH is basically extended to about 50m, there will be no more significant changes in Figure 12. Different from the migration characteristics of other components, the migration range of H^+ concentration was not too far away, which may be related to the active hydrogeochemical characteristics of H^+, which participated in more reactions during the migration and caused more consumption in Figure 13.

(Figure 12)

(Figure 13)

4.2.4 variation in other water chemical components

After one year of mining, the main law of the change of Ca^{2+} is that on the mining side of the mining area, the concentration was relatively high, and the concentration range can be from 0.01mol/Kg to 0.012mol/Kg. In the process of gradual diffusion to the periphery of the mining area, the concentration of Ca^{2+} was gradually from high to low and can exceed 0.012mol/Kg. This is due to the gradual dissolution of the carbonate minerals outside the mining area as the H^+ migrated and diffused outward. The concentration front appeared at about 150m to 180m away from the mining area, the concentration of Ca^{2+} decreased rapidly from 0.012mol/Kg to the background value. After one year of mining, the main rule of the change of Mg^{2+} is that on the mining side of the mining area, the concentration was relatively high, and the concentration can exceed 0.018mol/Kg. In the process of gradual diffusion to the periphery of the mining area, the concentration of Mg^{2+} gradually advanced, which was due to the gradual dissolution of Mg-containing carbonate minerals outside the mining area with the outward migration and diffusion of H^+. About 150m away from the mining area, a concentration front of Mg^{2+} appeared, and the concentration of Mg^{2+} decreased rapidly, From 0.018mol/Kg to the background value. After one year of mining, the main rule of Na^+ change is that on the mining side of the mining area, the concentration was relatively high, and the concentration can exceed 0.065mol/Kg. In the process of gradual diffusion to the periphery of the mining area, the concentration of Na^+ gradually advanced. This is due to the gradual dissolution of feldspar minerals and clay minerals containing Na^+ outside the mining area with the outward migration and diffusion of H^+. About 150m away from the mining area, there was a concentration front of Na^+, and the concentration of Na^+ decreased rapidly, from 0.065mol/Kg to the background value. The migration law of K^+ and Na^+ was relatively similar. After one year of mining, the main change law of K^+ is that on the mining side of the mining area, the concentration is relatively high, and the concentration can exceed 0.065mol/Kg. In the process of gradual diffusion to the periphery of the mining area, the concentration of K^+ gradually advanced, which was due to the gradual dissolution of feldspar minerals and clay minerals containing K^+ outside the mining area as the H^+ moved outward and diffused.

The concentration front of K^+ appeared around 150m away from the mining area, The K^+ concentration decreased rapidly from 0.065mol/Kg to the background value. The concentration of HCO_3^- was relatively low in the interior of the mining area due to the greater impact of pH, while it was relatively high in the outer area close to the pumping well, and gradually reduced in the far area. SO_4^{2-} is the ion with high concentration in the area, which was in the mining area, and can exceed 0.13mol/Kg at the maximum. The concentration was large, and the migration distance was far, which can exceed 120m. This should be noted. This is mainly because SO_4^{2-} is many implanted ions in the mining area, unlike the high consumption of H^+. Although some SO_4^{2-} was precipitated with minerals, some SO_4^{2-} migrated far due to the large injection amount. The concentration of AlO_2^- in the mining area was relatively low, while the concentration was relatively high from the outside of the mining area to about 150m. The concentration front was about 150m away from the mining area, and the overall concentration of Cl^- changed little. On the contour line, the concentration of Cl^- in the mining area was relatively low, caused by the fact that the concentration of Cl^- in the injection solution was slightly lower than the background value of groundwater, while the mineral solubility of Cl^- was generally strong, so Cl^- was rarely replenished by mineral dissolution, It could only be diluted by the injection solution, so its concentration was relatively low in the mining area, but because the concentration of Cl^- in the injection solution was also relatively high, it can only be said that the background value of groundwater was slightly low in Figure 14.

(Figure 14)

4.2.5 Dissolution and precipitation of other minerals

Gypsum is also one of the minerals in the mining process. When the leaching solution is injected into the formation, it will cause the dissolution of calcite. At the same time, due to the high concentration of SO_4^{2-}, there will be gypsum precipitation. It can be seen from the distribution diagram of gypsum volume fraction increase that the increase of gypsum mineral volume fraction is small near the injection well, while the increase of gypsum mineral volume fraction is large in the mining area far from the injection well. This is because the pH near the injection well is low, and the groundwater environment near the injection well is in a strong acidic state. Therefore, although there is a high concentration of Ca^{2+} and SO_4^{2-}, precipitation is not easy to occur. In the figure 15, the volume fraction of anhydrite increases slightly near the injection well, less than 0.5%, while near the pumping well, the volume fraction of anhydrite increases, with a maximum increase of more than 3.5%. Calcite is one of the main dissolved minerals in the study area and one of the sources of Ca^{2+} in the mining area. From the increase diagram of calcite volume fraction, calcite is in a dissolved state in the mining area, and the volume fraction decreases by 2.8%.

(Figure 15)

K-feldspar and orthoclase are two kinds of feldspar minerals, and their volume fraction change trend is relatively similar, but the change range of potassium feldspar is larger. Near the injection well, the volume fraction of feldspar minerals decreased significantly, and the volume fraction of potassium feldspar decreased by up to -9×10^{-6}, the volume fraction of feldspar can be reduced by -4.5×10^{-7}, with the volume fraction of feldspar

minerals away from the injection well decreasing, the trend was gradually weak in Figure16. The volume fraction of feldspar minerals showed a decreasing trend, indicating that these minerals were mainly dissolved near the mining area. The volume fraction of quartz minerals has little change. Hematite and pyrite are two kinds of iron-bearing minerals in the mining area. Hematite in the mining area was generally in the precipitation state, while pyrite was in the dissolution state. The iron content in the mining area was high, which was closely related to the dissolution and precipitation of iron-bearing minerals.

(Figure 16)

This simulation included five clay minerals, namely Ca-smectite, Na-smectite, illite, chlorite and kaolinite. Calcium-montmorillonite and sodium-montmorillonite were in the dissolution state during the extraction process, while the volume fraction of sodium-montmorillonite decreased more than that of Calcium-montmorillonite in Figure 17. Illite and chlorite were in both precipitation and dissolution states. Specifically, they were dissolved in the mining area near the acid solution injection, and gradually turned to precipitation with outward expansion, in which illite had a larger precipitation. However, kaolinite had always been in the precipitation state. The dissolution of montmorillonite minerals was greater near the injection well, and less far away from the injection well. Illite and chlorite were dissolved near the injection well and precipitated far away from the injection well. Kaolinite had the largest amount of precipitation only near the outer liquid injection well.

(Figure 17)

Muscovite minerals were added to the simulation as secondary minerals. From the simulation results, muscovite minerals mainly appeared in the center of the injection well, outside the mining area with a certain distance between the pumping well and the injection well, while in the middle of the seepage zone of the pumping well and the injection well, the volume integral number increased little in Figure 18. The volume distribution of siderite and ferridolomite was very similar. From the diagram of their volume fraction change, the two minerals mainly precipitate outside the mining area, and the precipitation trend gradually decreased from the edge of the mining area to the outside, indicating that these two minerals were greatly affected by the high salinity solution transferred from the mining area. In the internal area of the mining area, due to the strong acidity, siderite and ferridolomite were difficult to precipitate, When the solution in the mining area migrated and diffused to the outside, the pH gradually decreased, the saturation index of many ions is large, and precipitation occurred immediately. With the increase of migration distance, each ion gradually consumed, and the precipitation trend decreased.

(Figure 18)

4.2.6 Change of porosity and permeability structure of reservoir

The porosity and permeability is the important parameter of the reservoir. Izgec explored the porosity with significant high in rock permeability using classical porosity-permeability models[39], and granite showed different behavior in dependent of permeability and porosity[40].The changing trend of porosity and reservoir

permeability was relatively similar as shown in figure 19. The porosity increased near the injection well, with a maximum increase value of about 0.12. This is because the acid concentration near the injection well was relatively high, and the salinity of the injection fluid was relatively low, so the minerals near the injection well undergo relatively severe corrosion, while the porosity decreased near the pumping well, with a minimum decrease value of about 0.07. Although near the pumping well, the pH value was still acidic, but the acidity was weakened compared to that at the liquid injection well, and because the content of various ions in the groundwater was greatly increased, some minerals (such as gypsum) were still precipitated. The change trend of permeability was inversely proportional to the change trend of porosity, increasing at the injection well and decreasing at the pumping well.

(Figure 19)

5. Conclusions

In this study, the Bayanwula mining area is taken as the research object, and the reactive solute transport simulation in the process of in-situ leaching of uranium is carried out by selecting the extended strip area at the edge of the mining area as the simulation area. The model depicts the migration distance and change trend of uranium elements, H^+ and various hydro chemical components that may be involved in mining. At the same time, the model describes the migration distance and change trend of uraninite, feldspar minerals, clay minerals. The state of calcite and other minerals that may undergo dissolution and precipitation is described.

The research results show that calcite is the most easily dissolved mineral, and other minerals will have significant dissolution after the dissolution of calcite. Calcite is also one of the sources of gypsum precipitation. Due to the consumption of calcite, the leaching solution will gradually dissolve uranium ore, hematite, and other minerals. The uranium is basically limited in the mining area, and the trend of outward migration is very weak. The reaction property of H^+ is active, and the injection amount is also large. However, because it will react with various minerals in the mining area, its outward influence distance will not be too far, and the influence distance of K^+, Na^+, Ca^{2+}, Mg^{2+} and other major metal cations is relatively far with the development of the mining process, their concentration front is about 150m away from the outside of the mining area. At the same time, the dissolution area of minerals mainly occurs in the interior of the mining area, the approximate range is close to the coverage of H^+, which is also due to the hydraulic trap effect of the pumping and injection wells, and only a small amount of H^+ migrates to the periphery of the mining area. The simulation results show that uraninite is mainly dissolved near the injection well, but some uranium elements will precipitate out again with the migration to the pumping well. The main dissolved minerals include K-feldspar, oligoclase, pyrite, calcite, Na-smectite, and Ca-smectite. These minerals provide the main source of metal cations in the underground water of the mining area. Illite and chlorite dissolve inside the mining area and precipitate outside the mining area. Hematite as precipitation minerals, mainly precipitate inside the mining area. Siderite and ankerite are precipitated outside the mining area as precipitation minerals. Gypsum minerals are precipitated inside and outside the mining area. At the same time, in the process of mining, it will cause

changes of porosity and permeability structure of the mining area. Near the injection well, many minerals in the formation will dissolve, resulting in increased porosity and permeability, while in the vicinity of the pumping well, the mineral precipitation will lead to the reduction of porosity and permeability, which may cause the blockage of the pore channel, which will have a negative impact on the mining efficiency. The simulation results show that during the mining process, the scope of strong acidity of groundwater is principally in the mining area, while outside the mining area, due to the existence of many minerals, the acidic substances overflowing to the periphery of the mining area are neutralized, resulting in a rise of numerous elements.

Reference

[1] Connor R. The United Nations World Water Development Report 2015: water for a sustainable world. UNESCO Publishing, 2015

[2] Malik Muhammad Akhtar, Abida Dost Mohammad, Muhsan Ehsan, Rabia Akhtar, Jalil ur Rehman. Water Resources Of Balochistan, Pakistan-A Review. Arabian Journal of Geosciences, 2021, 14, 4: 1-16

[3] Manoj, S., Thirumurugan, M., & Elango, L. An integrated approach for assessment of groundwater quality in and around uranium mineralized zone, Gogi region, Karnataka, India. Arabian Journal of Geosciences, 2017, 10, 1-17.

[4] Raja, V., & Neelakantan, M. A. Application of GIS technique to address the uranium contamination in groundwater of a hard rock aquifer, South India. Geocarto International, 2022, 1-15.

[5] Sharma DA, Keesari T, Rishi MS, Pant D. A study on the role of hydrogeology on the distribution of uranium in alluvial aquifers of northwest India. Environ Monit Assess, 2018, 190(12): 1–21.

[6] Que Weimin, Tan Yahui, Zeng Yijun, Wang Shude. Reaction kinetics and material transport of in-situ leaching of uranium. Beijing: Atomic Energy Press, 2002, 191.

[7] Zhou Xitang, Que Weiming, Hu Eming, Zhou Quan, Su Xuebin Migration and sedimentation of uranium elements in in-situ leaching . Uranium Mining and Metallurgy, 2000, 19 (2): 78

[8] Peng Dingmao, Zhang Hongjun, Wang Shude Study on water-rock interaction of in-situ leaching of uranium. Journal of East China Geology, 2001, 24 (1): 41

[9] Gao Bo, Sun Zhanxue, Shi Weijun. Experimental study on characteristics and conditions of uranium migration during in-situ leaching . Geological Review, 2003, 49 (3): 316

[10] Huang Qunying. Analysis of solute migration and acidification process of acid in-situ leaching of a sandstone uranium mine. Non-ferrous metals (smelting part), 2015, (6): 50

[11] Huang Qunying, Zhou Yipeng, Liu Ke, Chen Meifang. The relationship between solute transport and solution percolation at the initial stage of acid leaching of a placer uranium ore in-situ leaching unit. Nonferrous Metals (Smelting Part), 2017, (6): 24

[12] Ilton, E.S., Haiduc, A., Cahill, C.L., Felmy, A.R., Mica surfaces stabilize pentavalent uranium. Inorg. Chem, 2005, 44, 2986–2988.

[13] Wan, J., Tokunaga, T.K., Brodie, E., Wang, Z., Zheng, Z., Herman, D., Hazen, T.C.,Firestone, M.K., Sutton, S.R., Reoxidation of bioreduced uranium under reducing conditions. Environ. Sci. Technol, 2005, 39, 6162–6169.

[14] Ginder-Vogel, M., Criddle, C.S., Fendorf, S., Thermodynamic constraints on the oxidation of biogenic UO_2 by Fe(III) (hydr)oxides. Environ. Sci. Technol, 2006, 40,3544–3550.

[15] Rai, D., Felmy, A.R., Ryan, J.L., Uranium(IV) hydrolysis constants and solubility product of UO_2 xH_2O(am). Inorg. Chem., 1990, 29: 260–264.

[16] Kate M.Campbell , et al. Biogeochemical aspects of uranium mineralization, mining, milling, and remediation. Applied Geochemistry, 2015, 57:206–235.

[17] Hsi, C. D., and D. Langmuir, Adsorption of uranyl onto ferric oxyhydroxides; application of the surface complexation site-binding model. Geochim. Cosmochim. Acta, 1985,49(9): 1931–1941.

[18] Waite, T. D., J. A. Davis, T. E. Payne, G. A. Waychunas, and N. Xu. Uranium (VI) adsorption to ferrihydrite; application of a surface complexation model, Geochim. Cosmochim. Acta, 1994, 58(24): 5465– 5478.

[19] Pabalan, R. T., D. R. Turner, F. P. Bertetti, and J. D. Prikryl, UraniumVI sorption onto selected mineral surfaces; key geochemical parameters, in Adsorption of Metals by Geomedia; Variables, Mechanisms, and Model Applications, edited by E. A. Jenne,1998, pp. 99–130, Elsevier, New York.

[20] Davis, J. A., T. E. Payne, and T. D. Waite, Simulating the pH and pCO_2 dependence of uranium (VI) adsorption by a weathered schist with surface complexation models, in Geochemistry of Soil Radionuclides, 2002, pp. 61– 86, Soil Sci. Soc. of Am., Madison, Wis.

[21] Steven B. Yabusaki, et al. Building conceptual models of field-scale uranium reactive transport in a dynamic vadose zone-aquifer-river system, WATER RESOURCES RESEARCH, VOL. 44, W12403, doi: 10. 1029/2007WR006617, 2008

[22] Sun Ming. Laboratory experimental study on in-situ leaching and hydrometallurgy of a sandstone uranium mine. East China University of Technology(Nanchang), 2012.

[23] Jiao Xueran, Sun Zhanxue, etc Study on the blocking mechanism of in-situ leaching of a high salinity sandstone-type uranium mine. Non-ferrous metals (smelting part), 2013(8):25-28.

[24] Su Xuebin, et al. Experimental study on CO_2 in-situ leaching of uranium from a uranium mine in Xinjiang. Uranium Mining and Metallurgy, 2005, 24(3):129-133

[25] Wen Zhenqian. Impurity ion Precipitation and Its Effect on Uranium Precipitation during Acid In-situ Leaching . Uranium Mining and Metallurgy, 2015, 34(3): 171-177

[26] Zhenghe Qiuye., In-situ leaching condition test and hydrodynamic simulation of in-situ leaching site at Bayanwula uranium mine. East China University of Technology (Nanchang), 2017.

[27] Chen Wanli. Study on the mechanism of chemical plugging of ore layers in the in-situ leaching process of Bayanwula uranium mine. East China University of Technology (Nanchang), 2014.

[28] Jiao Youjun, Shi Xiaoqing, Wu Jichun, et al. Numerical simulation of adsorption reaction and transport of hexavalent uranium in groundwater based on PHT3D, Groundwater, 2015 (2): 8-10

[29] Jiao Youjun, Shi Xiaoqing, Wu Jichun. Comparison of several adsorption reaction transport models of hexavalent uranium in the underground aquifer of uranium tailings pond leakage. Journal of Environmental Science, 2015, 35 (10): 3193-3201.

[30] Chen Qianiqian. Discussion on numerical simulation of acid in-situ leaching of uranium based on reactive solute transport. East China University of Technology (Nanchang), 2020.

[31] Xu, T., Pruess, K. Modeling multiphase non-isothermal fluid flow and reactive geochemical

transport in variably saturated fractured rocks: 1, Methodology. Am. J. Sci. 2001,301, 16–33.

[32] Xu, T., Spycher, N., Sonnenthal, E., Zhang, G., Zheng, L.,Pruess, K. TOUGHREACT Version 2.0: A simulator for subsurface reactive transport under non-isothermal multiphase flow conditions. Comput. Geosci. 2011, 37, 763–774.

[33] Pruess, K., Oldenburg, C., Moridis, G. TOUGH2 user's guide,version 2.0. Lawrence Berkeley Laboratory Report LBL-43134,Berkeley, CA,1999, p. 192.

[34] Xu, T., J. A. Apps & K. J. J. o. G. R. S. E. Pruess, Reactive geochemical transport simulation to study mineral trapping for CO2 disposal in deep saline arenaceous aquifers. 2003, 108.

[35] Xu T， Sonnenthal E, & Bodavarsson G. A reaction-transport model for non-isothermal multiphase reactive geochemical transport in variably saturated geologic media：Application to geothermal injectivity and CO_2 geological sequestration. Computers& Geosciences，2006，32（2）：145-165

[36] Van Genuchten, M.Th. A Closed-Form Equation for Predicting the Hydraulic Conductivity of Unsaturated Soils, Soil Sci. Soc. , 1980, Vol. 44, pp. 892 - 898.

[37] Mualem, Y. A New Model for Predicting the Hydraulic Conductivity of Unsaturated Porous Media, Water Resour. Res., 1976,Vol. 12, No. 3, pp. 513 - 522.

[38] Corey, A.T. The Interrelation Between Gas and Oil Relative Permeabilities, Producers Monthly, 1954,38-41.

[39] Izgec, O., Demiral, B., Bertin, H., & Akin, S. CO_2 injection into saline carbonate aquifer formations I. Transport in porous media, 2008, 72, 1-24.

[40] Jia, C. J., Xu, W. Y., Wang, H. L., Wang, R. B., Yu, J., & Yan, L. Stress dependent permeability and porosity of low-permeability rock. Journal of Central South University, 2017, 24(10), 2396-2405.

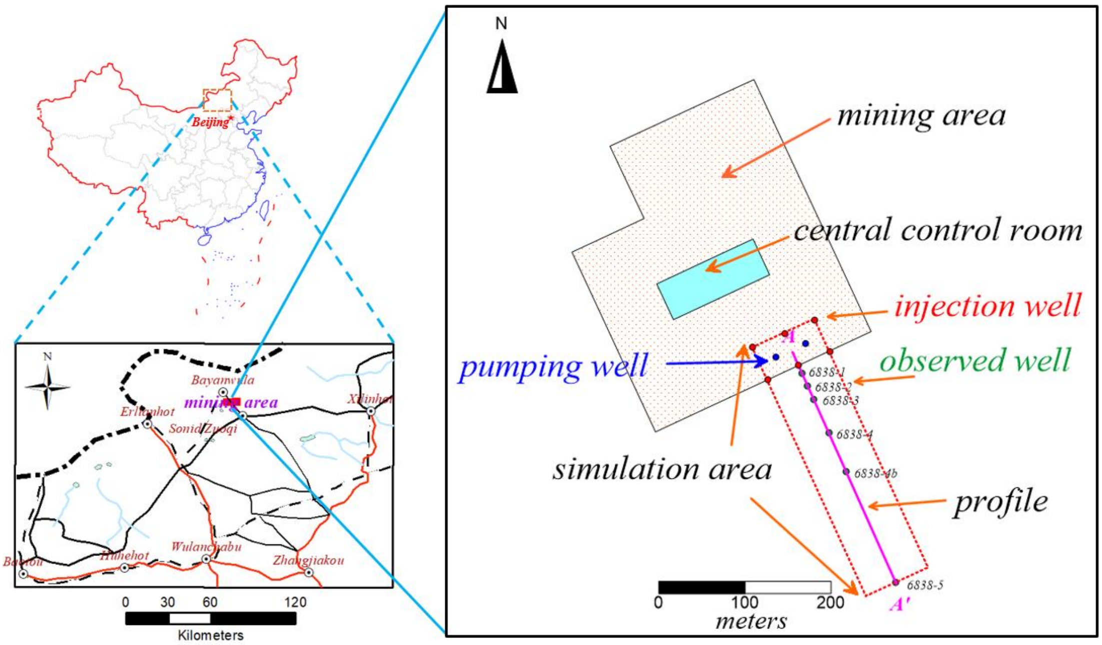

Figure 1. Traffic location map of Bayanwula mining area

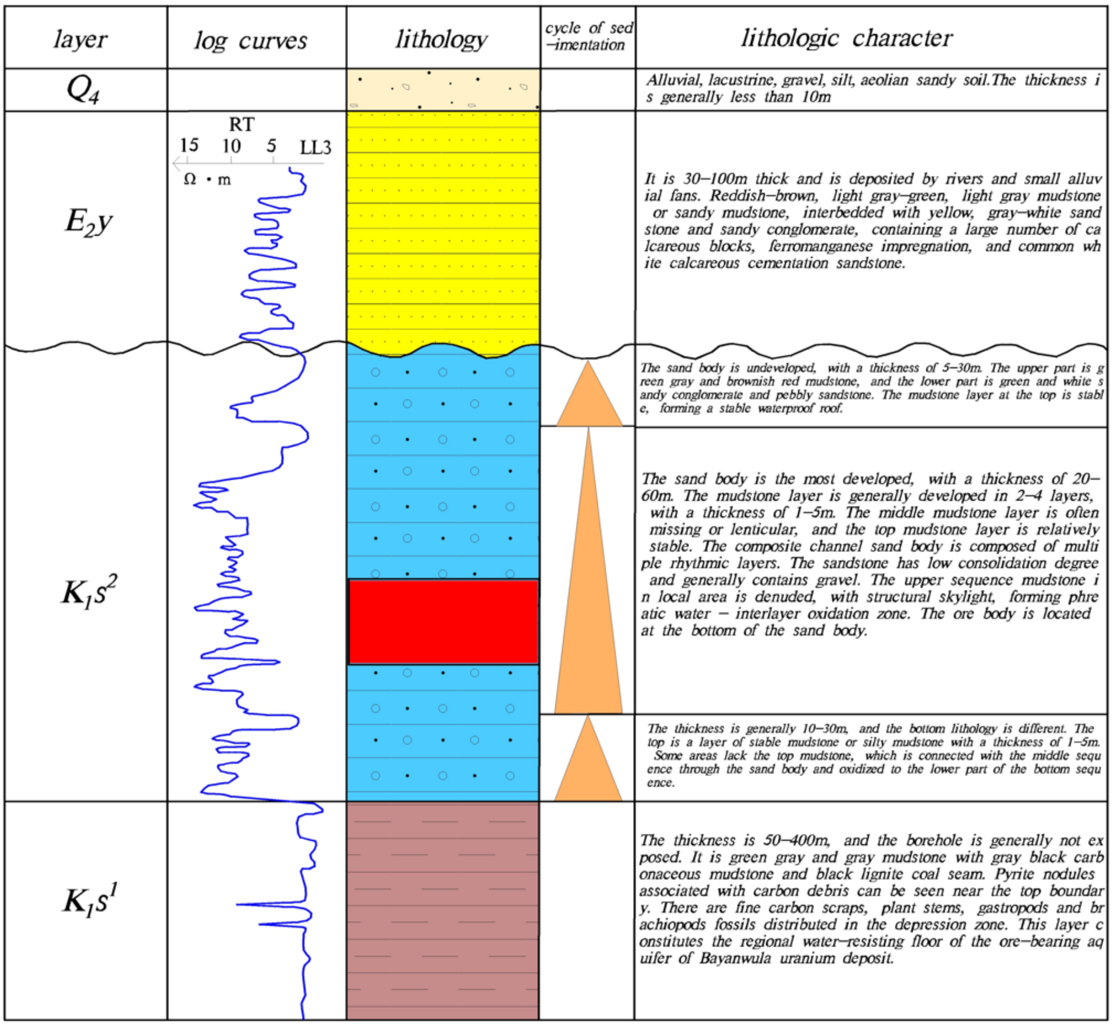

Figure 2 . Vertical sequence diagram of strata in the study area

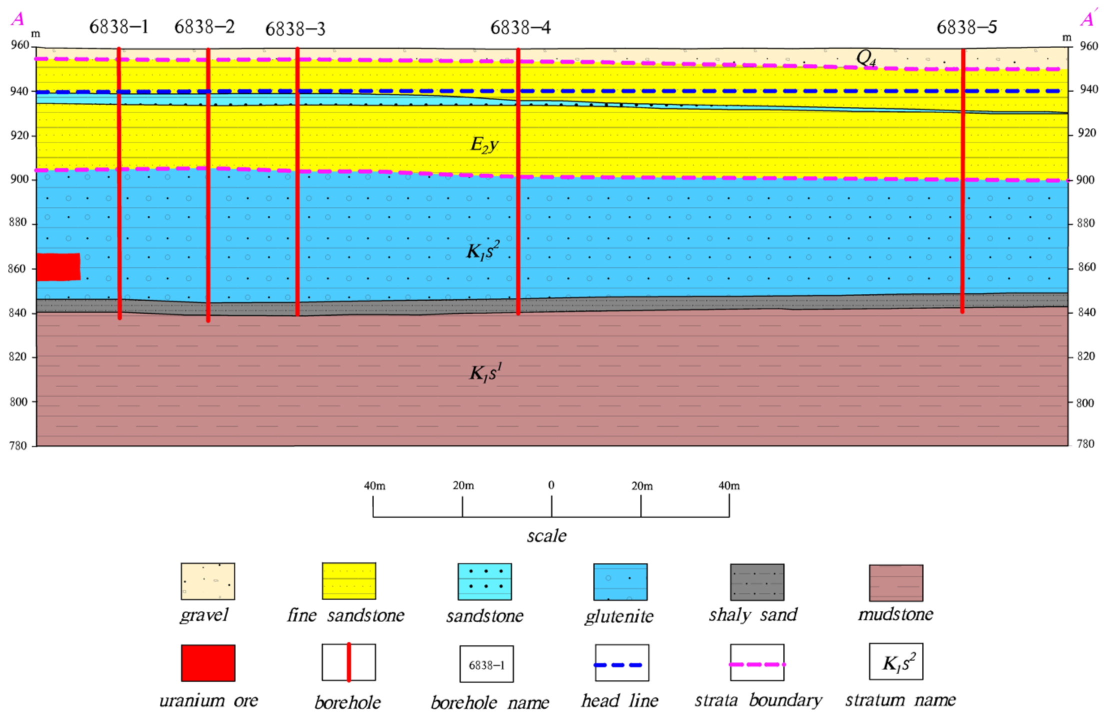

Figure 3. Typical section of the study area

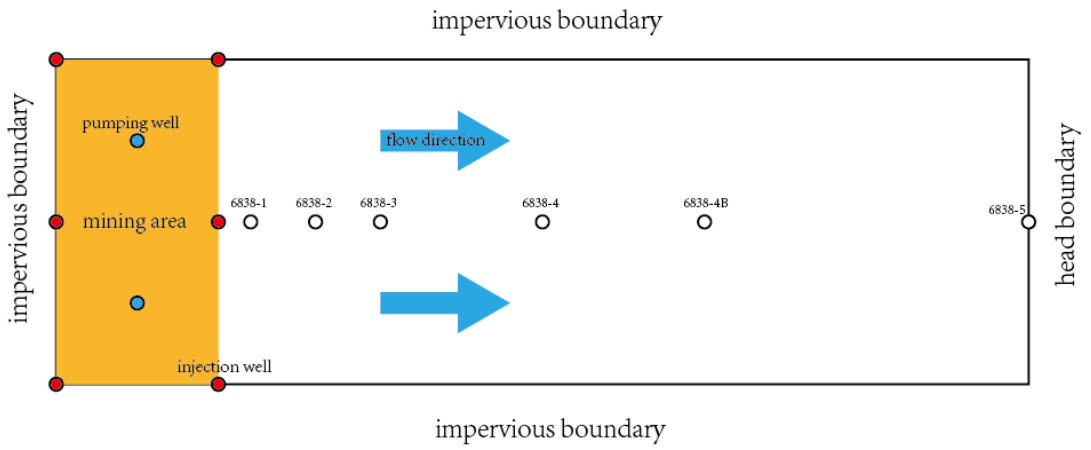

Figure 4. Conceptual model of reactive transport

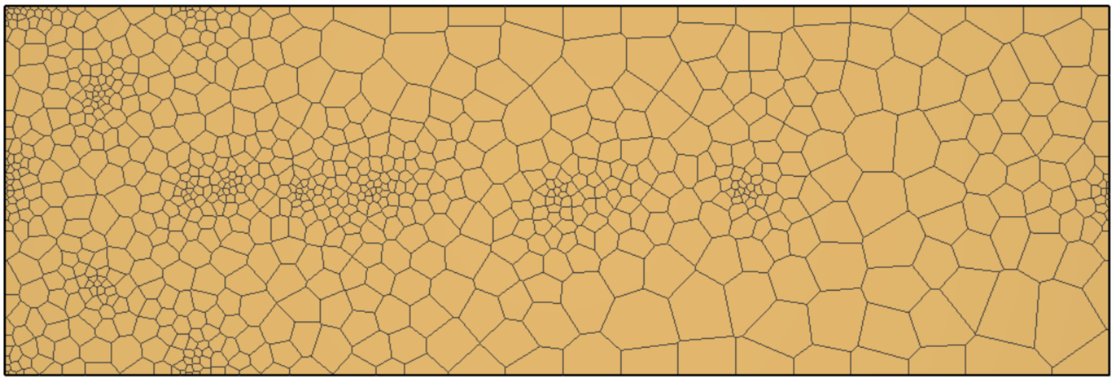

Figure 5. plane diagram of mesh

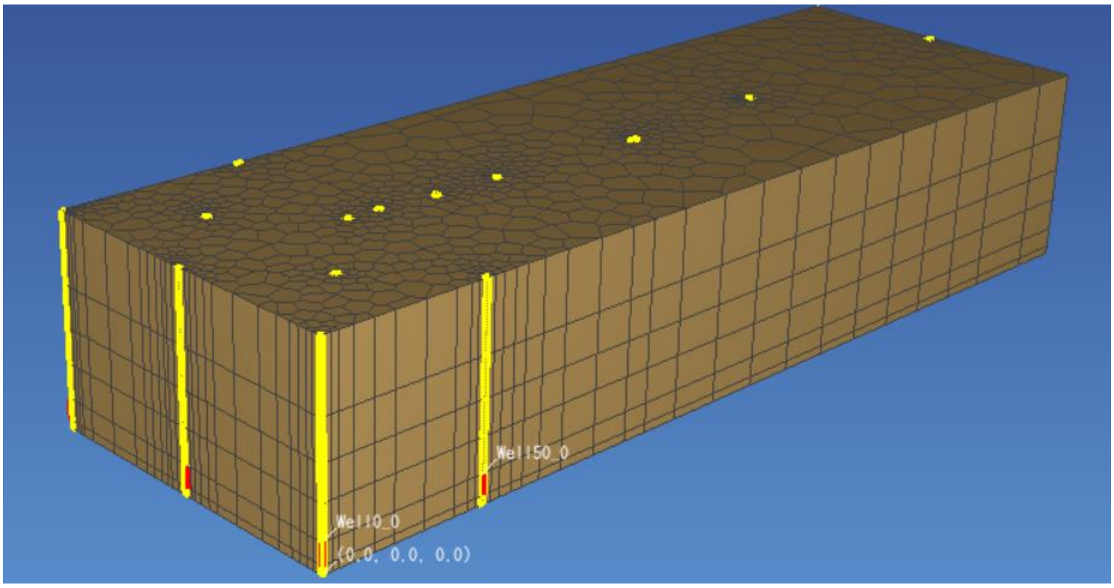

Figure 6. 3D structure diagram of mesh

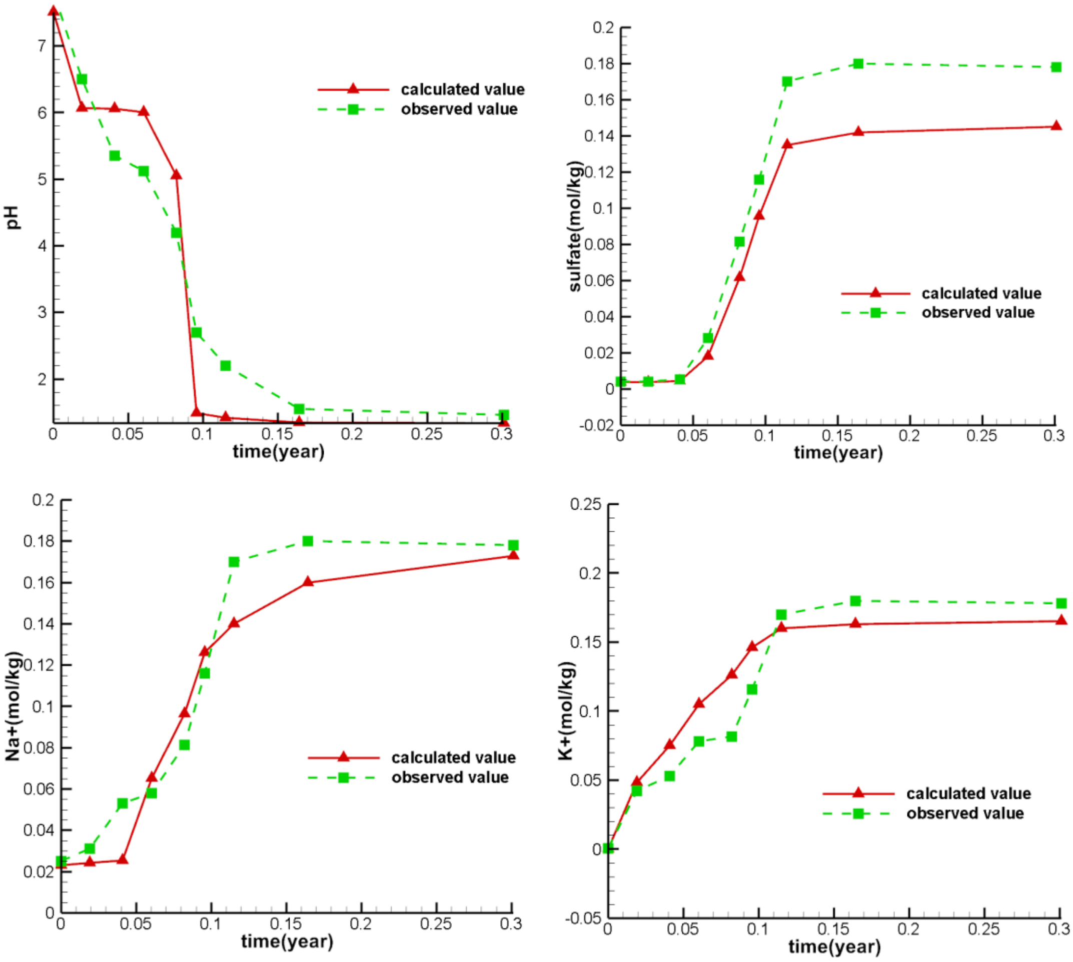

Comparison between observed and simulated values of 6838-1 hole test index

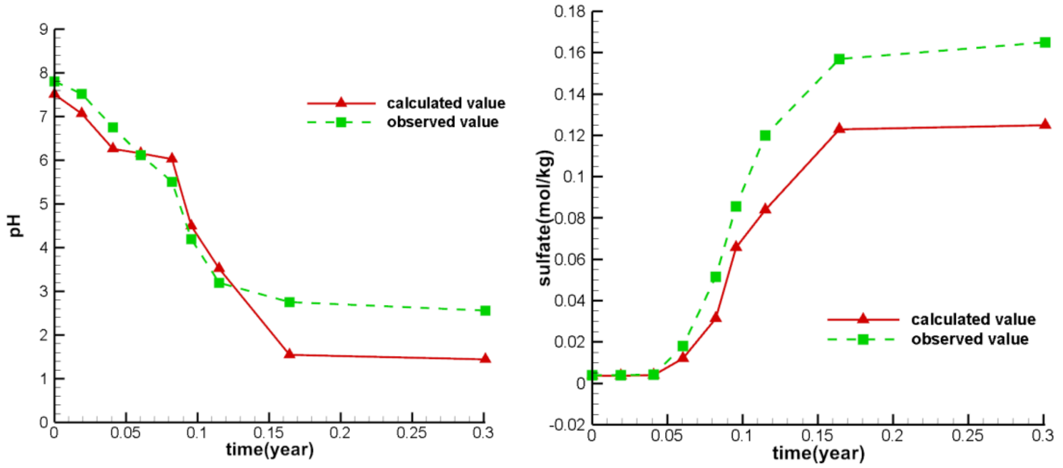

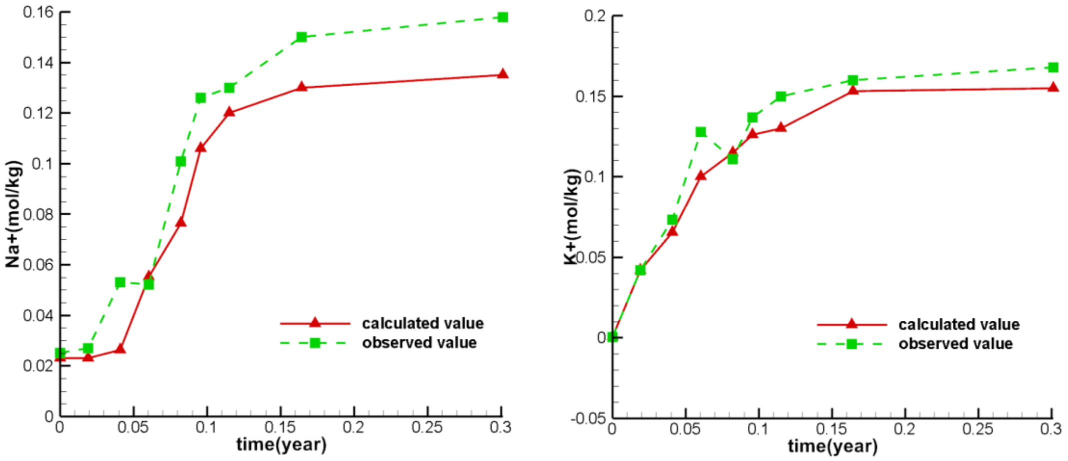

Comparison between observed and simulated values of 6838-2 hole test index

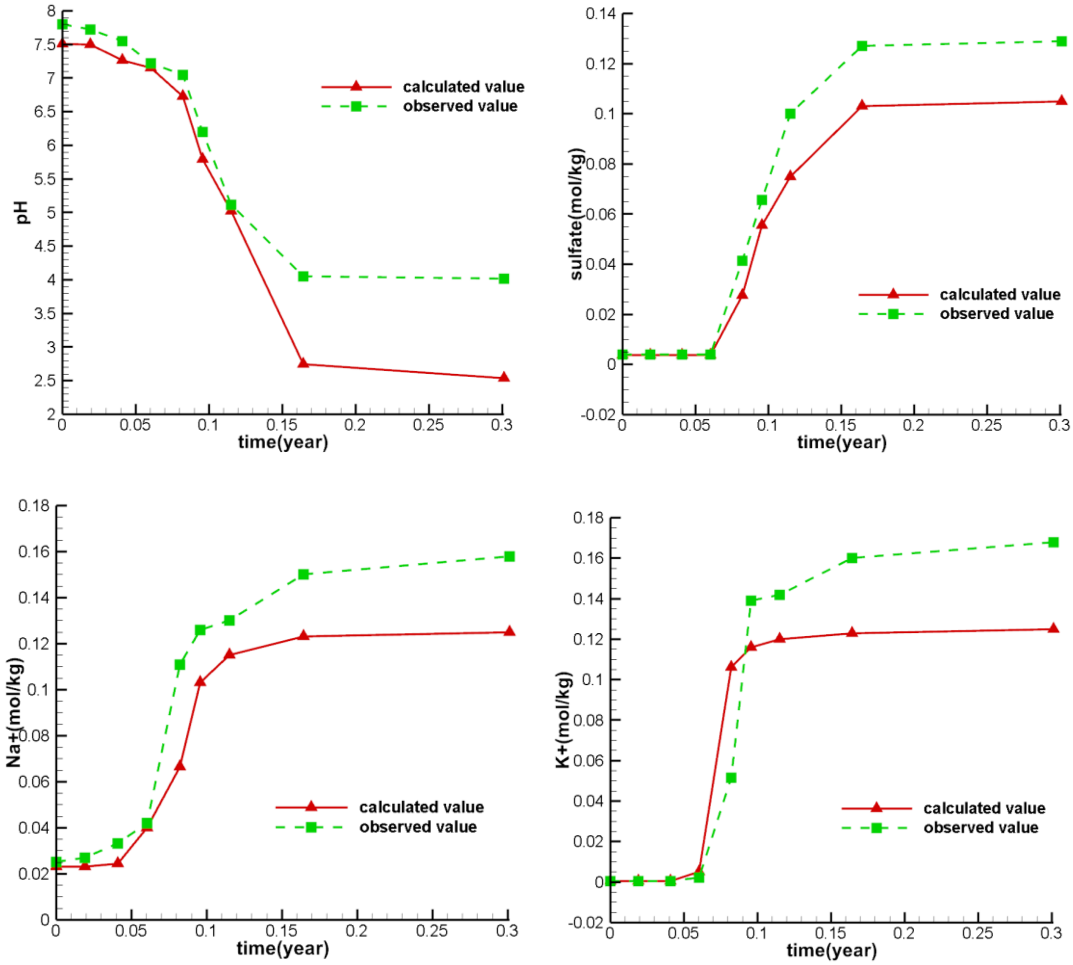

Comparison between observed and simulated values of 6838-3 hole test index

Figure 7.   Comparison between simulated and measured values

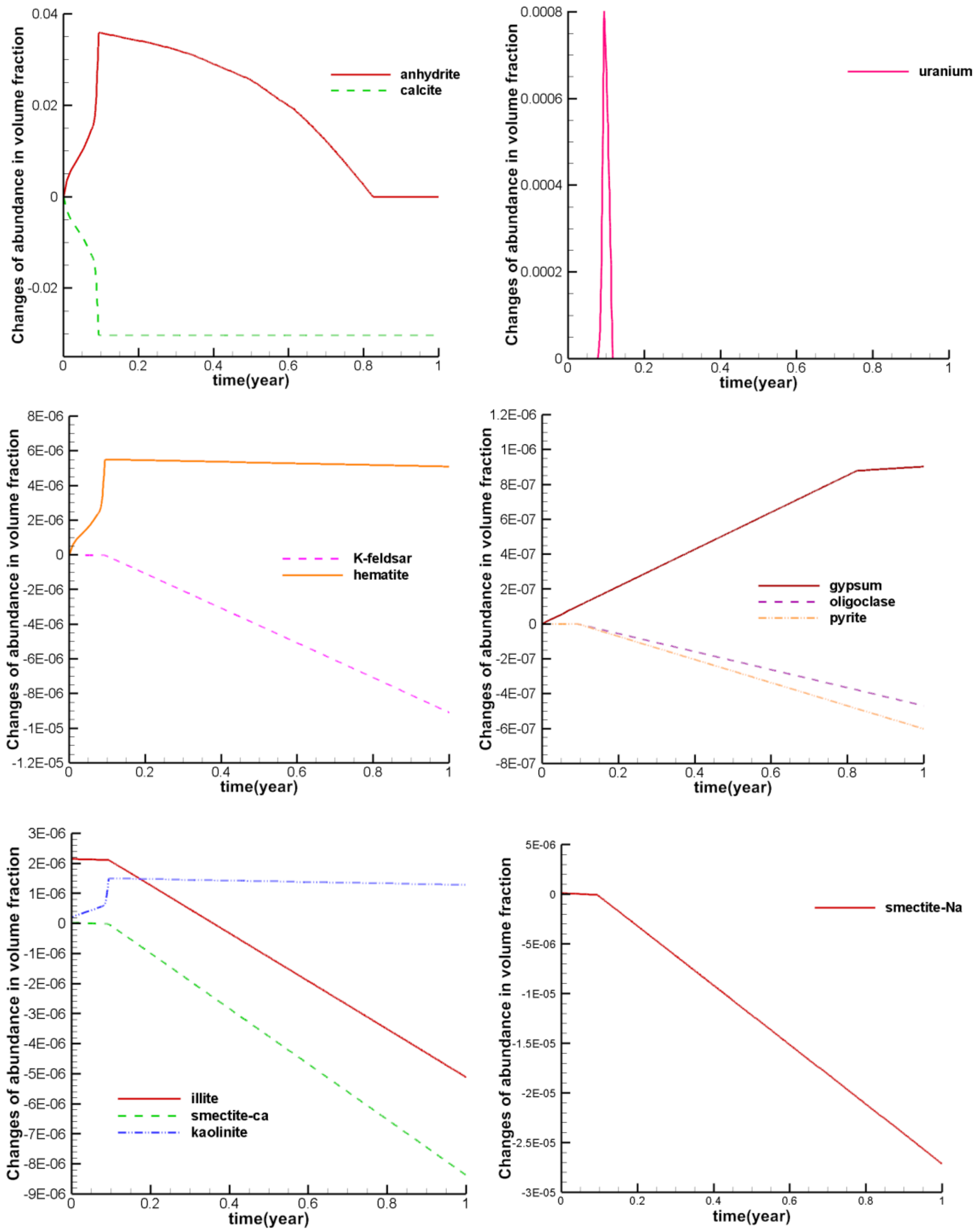
Changes of abundance in volume fraction
time(year)
anhydrite
calcite
uranium
K-feldsar
hematite
gypsum
oligoclase
pyrite
illite
smectite-ca
kaolinite
smectite-Na

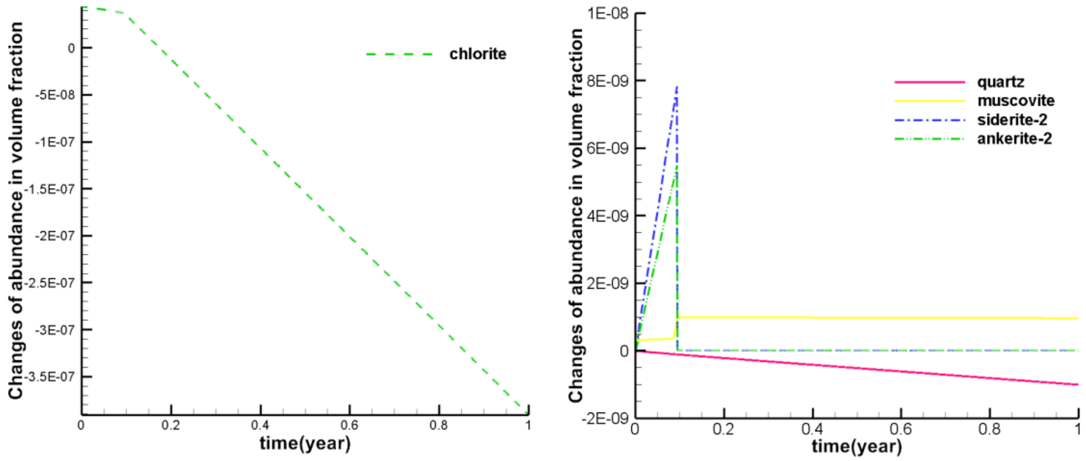

Figure 8 . The mineral volume fraction changes at observed well 6838-1

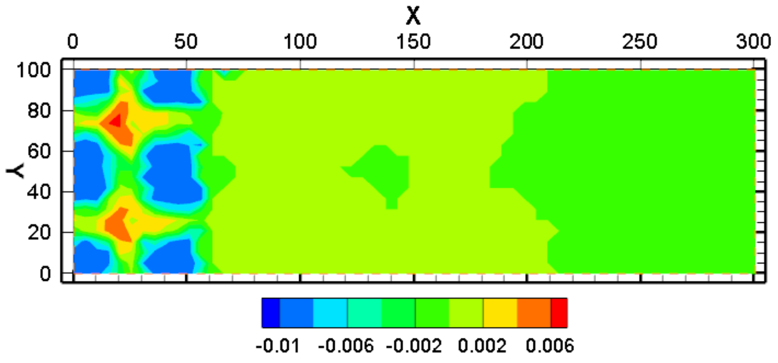

Figure 9. The volume fraction change of uranium

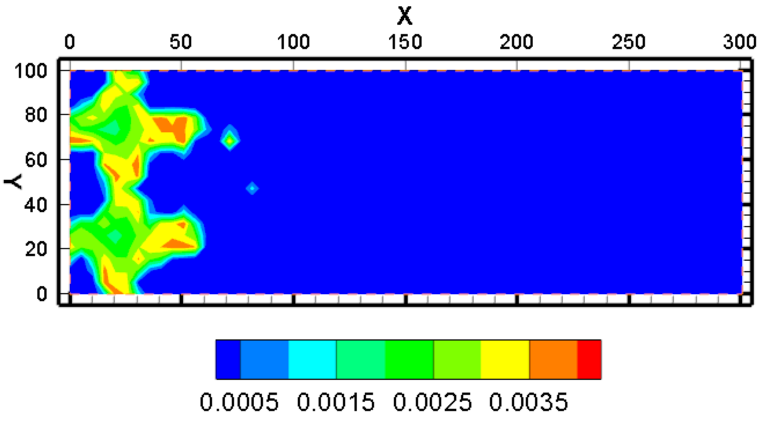

Figure 10. The concentration distribution of UO22+

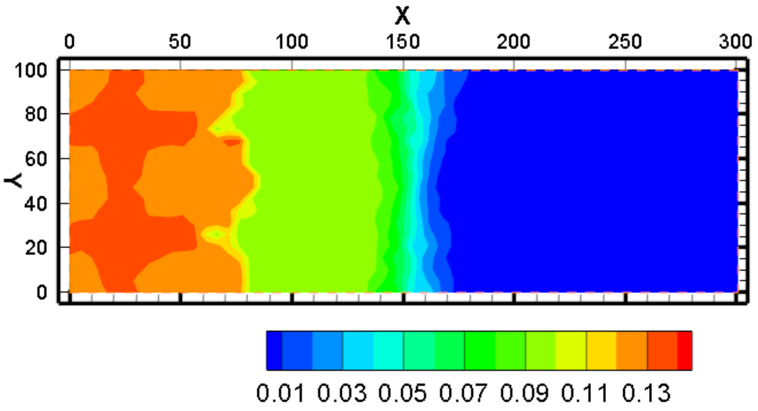

Figure 11. The concentration distribution of SO_4^{2-}

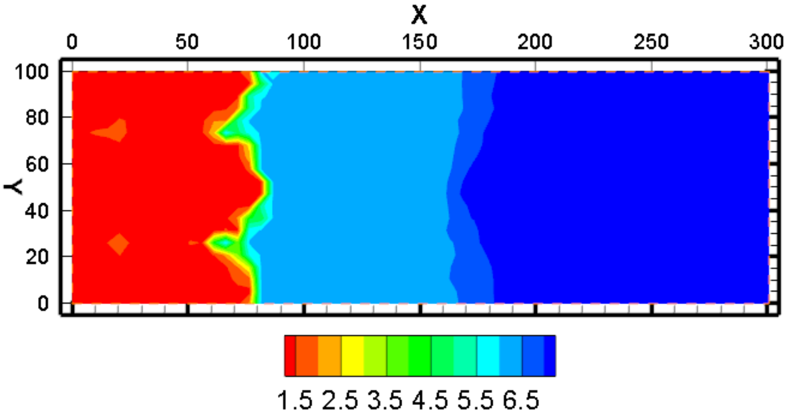

Figure 12. The distribution of pH value

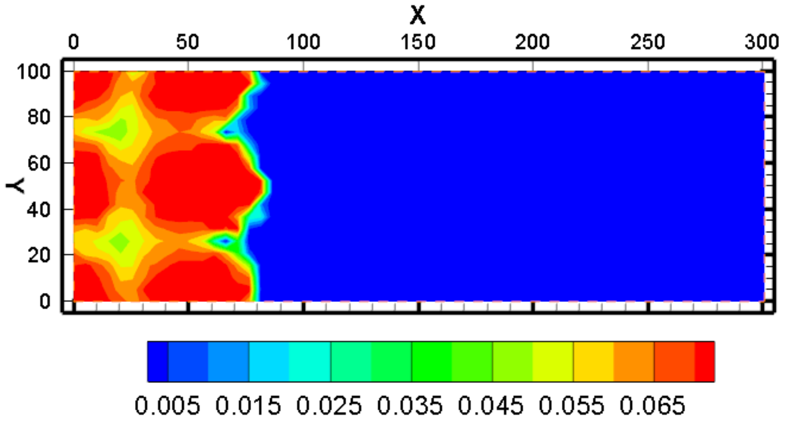

Figure 13. The concentration distribution of H^+

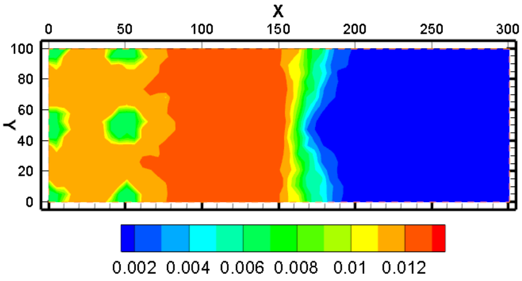

Concentration distribution graph of Ca^{2+}

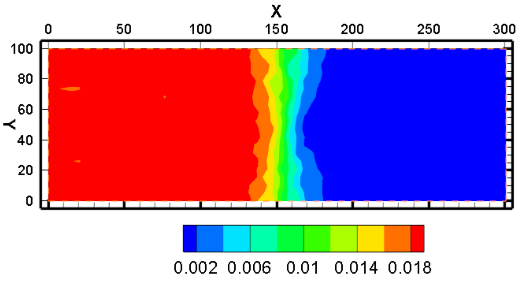

Concentration distribution graph of Mg^{2+}

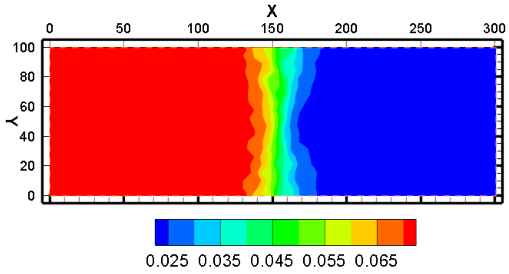

Concentration distribution graph of Na^{+}

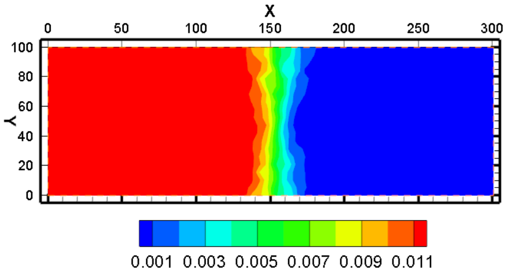

Concentration distribution graph of K^{+}

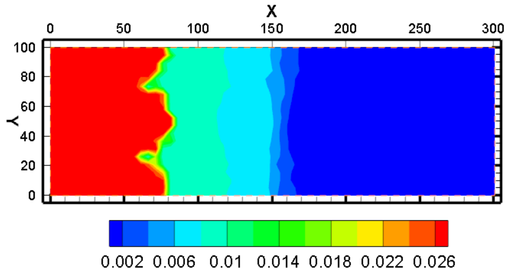

Concentration distribution graph of Fe^{2+}

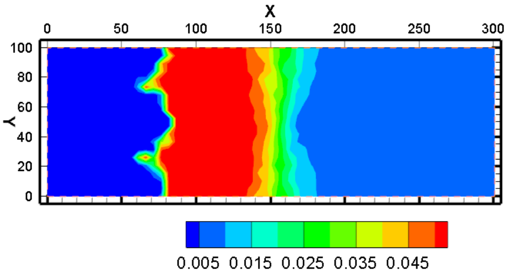

Concentration distribution graph of HCO_3^{-}

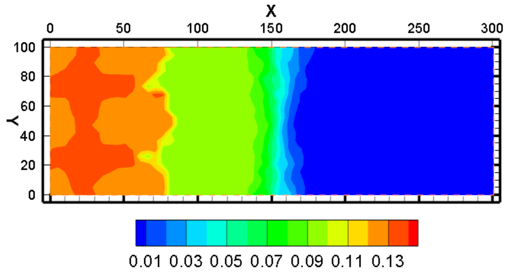

Concentration distribution graph of SO_4^{2-}

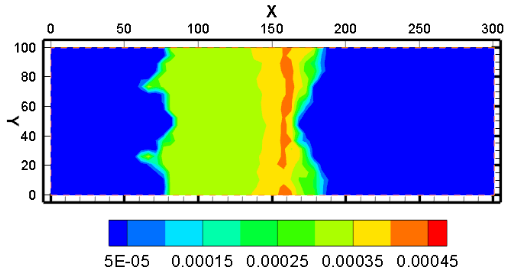

Concentration distribution graph of AlO_4^{-}

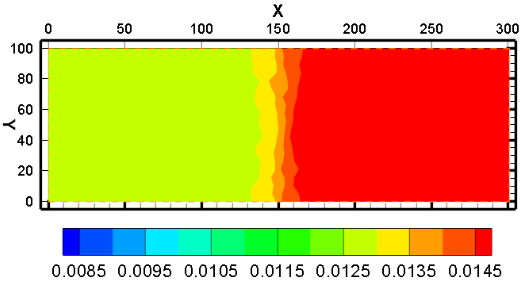

Concentration distribution graph of Cl^{-}

Figure 14. Concentration distribution graph of selected parameters

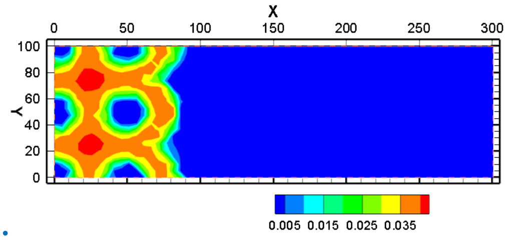

Volume fraction change of anhydrite

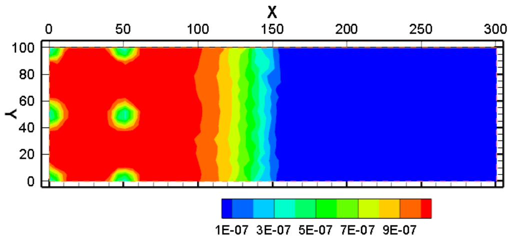

Volume fraction change of gypsum

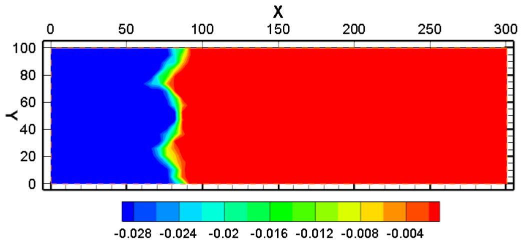

Volume fraction change of calcite

Figure 15. Volume fraction change of anhydrite, gypsum and calcite

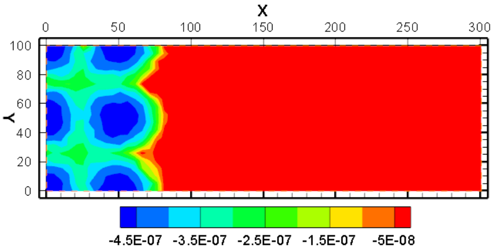

Volume fraction change of oligoclase

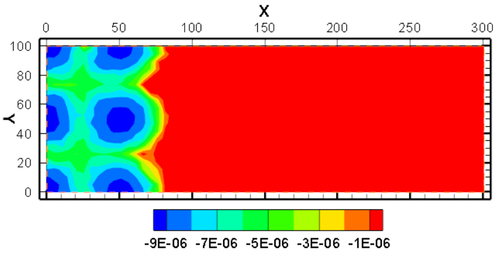

Volume fraction change of K-feldspar

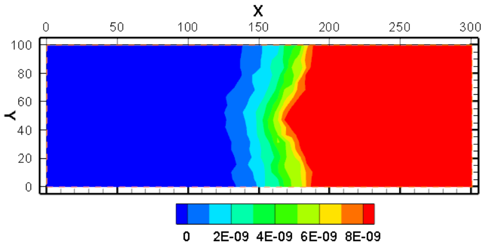

Volume fraction change of quartz

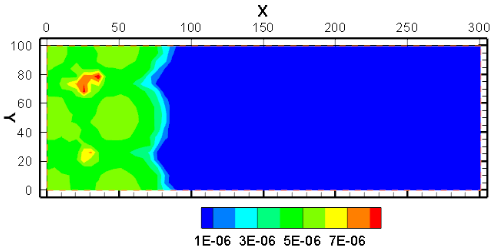

Volume fraction change of hematite

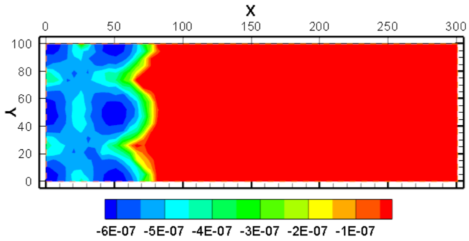

Volume fraction change of pyrite

Figure 16. Volume fraction change of various minerals

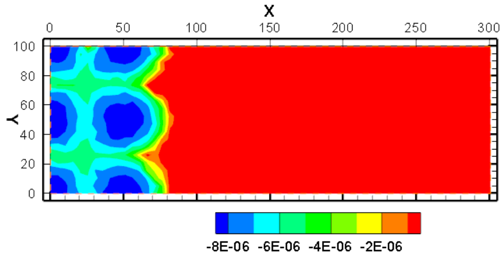

volume fraction change of Ca-smectite

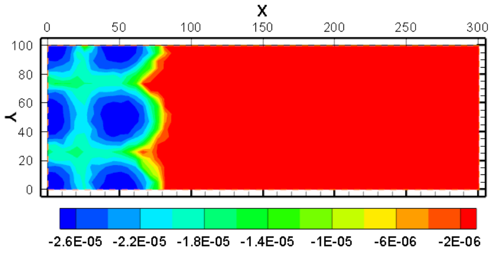

volume fraction change of Na-smectite

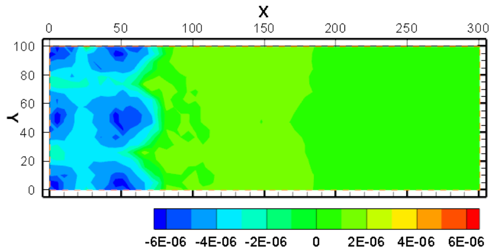

Volume fraction change of illite

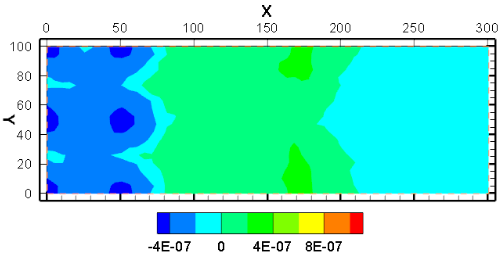

Volume fraction change of chlorite

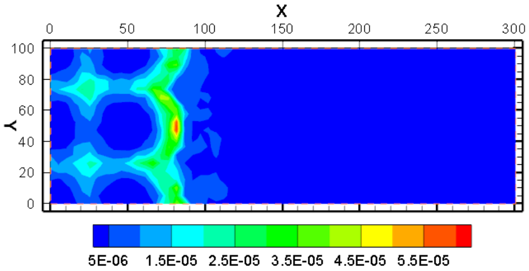

Volume fraction change of gaolinte

Figure 17. Volume fraction change of various minerals

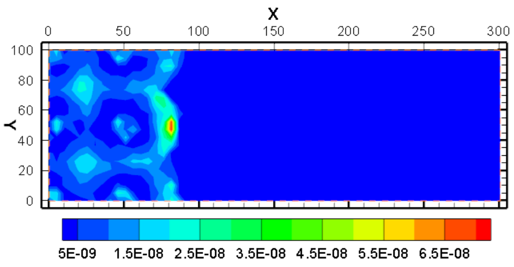

Volume fraction change of muscovite

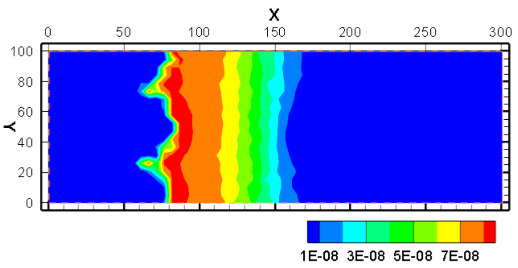

Volume fraction change of siderite

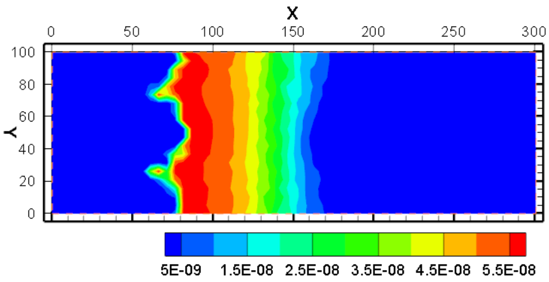

Volume fraction change of ankerite

Figure 18. Volume fraction change of various minerals

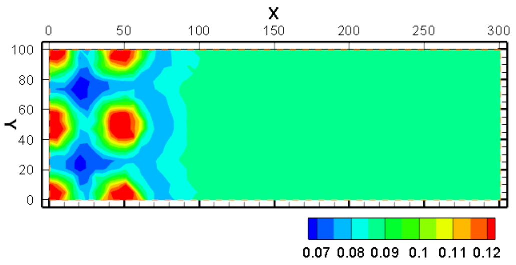

Change of porosity

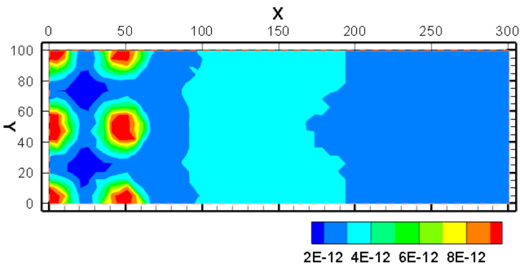

Change of permeability.

Figure 19. Change of porosity and permeability
