# Supplementary material for: Variation of groundwater and mineral composition of in situ leaching uranium in Bayanwula mining area, China
Source: PLoS One. 2024 Jul 12;19(7):e0303595. doi: 10.1371/journal.pone.0303595 (PMC11244825; doi:10.1371/journal.pone.0303595)
Supplement: Attachment — Submitted filename: Response_Review Comments_PONE-D-23-09655.docx [file pone.0303595.s002.docx]

To,

The Editor-in-Chief, PLOS ONE,

Subject: Reply to comments and submission of Manuscript Ref.: PONE-D-23-09655

Entitled: **Assessment of groundwater quality with reference to minerals in situ leaching uranium in Bayanwula mining area, China**

**Dear Editor,**

We appreciate the time and efforts of the editor and referees in reviewing this manuscript. We have carefully addressed all the issues indicated in the review report and believe that the revised version can meet the journal publication requirements.

To make the re-review process easy, we applied track-changes in the revised paper indicates changes are done according to comments. This response letter contains response of each comment.

Regards

**Dr.Malik Muhammad Akhtar**

PONE-D-23-09655R1
Assessment of groundwater quality with reference to minerals in situ leaching uranium in Bayanwula mining area, China
Professor Malik Muhammad Akhtar

Dear Dr. Akhtar,

We've checked your submission and before we can proceed, we need you to address the following issues:

1. We note that your uploaded Cover Letter is not a Cover Letter but a Response to Reviewers file. It is important that you include a cover letter with your manuscript. Please ensure that this letter is addressed specifically to PLoS ONE. Please also include
* why this manuscript is suitable for publication in PLoS ONE.
* how does your paper provide a worthwhile addition to the scientific literature?
* how does your paper relate to previously published work?
* which types of scientists do you believe will be most interested in your study?

Please also note that the Cover Letter and Response to Reviewers file should be uploaded as two separate files.

2. Please note that funding information should not appear in the Acknowledgments section or other areas of your manuscript. We will only publish funding information present in the Funding Statement section of the online submission form. Please remove any funding-related text from the manuscript.

3. Please amend the title either on the online submission form or in your manuscript so that they are identical.

4. Please amend your authorship list in your manuscript file to include author Wang Quanrong.

5. We note your Data Availability statement: "Data available on request"

PLOS journals require authors to make all data necessary to replicate their study’s findings publicly available without restriction at the time of publication. When specific legal or ethical restrictions prohibit public sharing of a data set, authors must indicate how others may obtain access to the data.

Please address the following:
a) At this time disclose the reason for this data restriction and provide a point of contact, preferably an email, for the ethical body mandating the restriction of this data. Please note that this point of contact cannot be an author of this study.

b) If there are no restrictions on this dataset, please provide your data by uploading it as a Supporting Information file or depositing it in a stable repository.

We hope to hear from you soon.

6. Regarding the potential copyright of figure 1, please respond to the following prompts:

A. For the above figures, were any shapefiles or basemaps obtained? If so, where were they obtained? Please provide any relevant links as well as any relevant copyright information.

B. For the above figures, were any external data utilized in the creation of these figures? If so, where were they obtained? Please inform us if the data was collected or produced by the authors. Please also provide any relevant links as well as any relevant copyright information.

C. Was any software (e.g. ArcGIS) used in the creation of these figures?

We look forward to your responses.

We've returned your manuscript to your account. Please resolve these issues and resubmit your manuscript within 21 days. If you need more time, please email the journal office at [plosone@plos.org](mailto:plosone@plos.org). We are happy to grant extensions of up to one month past this due date. If we do not hear from you within 21 days, we will withdraw your manuscript.

Please log on to PLOS Editorial Manager at <https://www.editorialmanager.com/pone/> to access your manuscript. You will find your manuscript in the 'Submissions Sent Back to Author' link under the New Submissions menu. Be sure to remove your previous manuscript file if you are uploading a new file in response to these requests. After you've made the changes requested above, please be sure to view and approve the revised PDF after rebuilding the PDF to complete the resubmission process.

We are requesting these changes to comply with the PLOS ONE submission guidelines (<https://journals.plos.org/plosone/s/submission-guidelines>). Please note that we won't send your manuscript for review until you have resolved the above requests.

Thank you for submitting your work to PLOS ONE and supporting our mission of Open Science.


Kind regards,

Rose Ann Joyce Sagun Puetes
PLOS ONE

PONE-D-23-09655

Assessment of groundwater quality with reference to minerals in situ leaching uranium in Bayanwula mining area, China

PLOS ONE

Dear Dr. Akhtar,

Thank you for submitting your manuscript to PLOS ONE. After careful consideration, we feel that it has merit but does not fully meet PLOS ONE’s publication criteria as it currently stands. Therefore, we invite you to submit a revised version of the manuscript that addresses the points raised during the review process.

Please submit your revised manuscript by Jun 25 2023 11:59PM. If you will need more time than this to complete your revisions, please reply to this message or contact the journal office at [plosone@plos.org](mailto:plosone@plos.org). When you're ready to submit your revision, log on to <https://www.editorialmanager.com/pone/> and select the 'Submissions Needing Revision' folder to locate your manuscript file.

Please include the following items when submitting your revised manuscript:

- A rebuttal letter that responds to each point raised by the academic editor and reviewer(s). You should upload this letter as a separate file labeled 'Response to Reviewers'.
- A marked-up copy of your manuscript that highlights changes made to the original version. You should upload this as a separate file labeled 'Revised Manuscript with Track Changes'.
- An unmarked version of your revised paper without tracked changes. You should upload this as a separate file labeled 'Manuscript'.

If you would like to make changes to your financial disclosure, please include your updated statement in your cover letter. Guidelines for resubmitting your figure files are available below the reviewer comments at the end of this letter.

If applicable, we recommend that you deposit your laboratory protocols in [protocols.io](http://protocols.io/) to enhance the reproducibility of your results. Protocols.io assigns your protocol its own identifier (DOI) so that it can be cited independently in the future. For instructions see: <https://journals.plos.org/plosone/s/submission-guidelines#loc-laboratory-protocols>. Additionally, PLOS ONE offers an option for publishing peer-reviewed Lab Protocol articles, which describe protocols hosted on [protocols.io](http://protocols.io/). Read more information on sharing protocols at <https://plos.org/protocols?utm_medium=editorial-email&utm_source=authorletters&utm_campaign=protocols>.

We look forward to receiving your revised manuscript.

Kind regards,

Vasanthavigar Murugesan, M.Sc., Ph.D.,<https://orcid.org/my-orcid?orcid=0000>
Academic Editor
PLOS ONE

7. We note that Figure 1 in your submission contain [map/satellite] images which may be copyrighted. All PLOS content is published under the Creative Commons Attribution License (CC BY 4.0), which means that the manuscript, images, and Supporting Information files will be freely available online, and any third party is permitted to access, download, copy, distribute, and use these materials in any way, even commercially, with proper attribution. For these reasons, we cannot publish previously copyrighted maps or satellite images created using proprietary data, such as Google software (Google Maps, Street View, and Earth). For more information, see our copyright guidelines: <http://journals.plos.org/plosone/s/licenses-and-copyright>.
 
We require you to either (1) present written permission from the copyright holder to publish these figures specifically under the CC BY 4.0 license, or (2) remove the figures from your submission:
 
a. You may seek permission from the original copyright holder of Figure 1 to publish the content specifically under the CC BY 4.0 license.  
 
We recommend that you contact the original copyright holder with the Content Permission Form (<http://journals.plos.org/plosone/s/file?id=7c09/content-permission-form.pdf>) and the following text:
“I request permission for the open-access journal PLOS ONE to publish XXX under the Creative Commons Attribution License (CCAL) CC BY 4.0 (<http://creativecommons.org/licenses/by/4.0/>). Please be aware that this license allows unrestricted use and distribution, even commercially, by third parties. Please reply and provide explicit written permission to publish XXX under a CC BY license and complete the attached form.”
 
Please upload the completed Content Permission Form or other proof of granted permissions as an "Other" file with your submission.
 
In the figure caption of the copyrighted figure, please include the following text: “Reprinted from [ref] under a CC BY license, with permission from [name of publisher], original copyright [original copyright year].”
 
b. If you are unable to obtain permission from the original copyright holder to publish these figures under the CC BY 4.0 license or if the copyright holder’s requirements are incompatible with the CC BY 4.0 license, please either i) remove the figure or ii) supply a replacement figure that complies with the CC BY 4.0 license. Please check copyright information on all replacement figures and update the figure caption with source information. If applicable, please specify in the figure caption text when a figure is similar but not identical to the original image and is therefore for illustrative purposes only.
The following resources for replacing copyrighted map figures may be helpful:
 
USGS National Map Viewer (public domain): <http://viewer.nationalmap.gov/viewer/>
The Gateway to Astronaut Photography of Earth (public domain): <http://eol.jsc.nasa.gov/sseop/clickmap/>
Maps at the CIA (public domain): <https://www.cia.gov/library/publications/the-world-factbook/index.html> and <https://www.cia.gov/library/publications/cia-maps-publications/index.html>
NASA Earth Observatory (public domain): <http://earthobservatory.nasa.gov/>
Landsat: <http://landsat.visibleearth.nasa.gov/>
USGS EROS (Earth Resources Observatory and Science (EROS) Center) (public domain): [http://eros.usgs.gov/#](http://eros.usgs.gov/)
Natural Earth (public domain): <http://www.naturalearthdata.com/>

Response：Figure-1 has been modified as per comments.

8. Please clarify Table 1 "Table 1. Well flow rate." in page "7" and Table 1 "Table 1 Secondary mineral in simulation" in page s"10 and 11" .

Response： Thanks for your comment. We have modified this as per comments.

Additional Comments From Editor

1. We note that several of your files are duplicated on your submission. Please remove any unnecessary or old files from your revision, and make sure that only those relevant to the current version of the manuscript are included.

Response: Additional files will be removed before submission

2. In your Methods section, please provide additional information regarding the permits you obtained for the work. Please ensure you have included the full name of the authority that approved the field site access and, if no permits were required, a brief statement explaining why.

Response: The project is a collaboration between China University of Geosciences (Wuhan) and Beijing Research Institute of Chemical Industry and Metallurgy. The research work has been approved by these units. In this study, all the materials were collected and investigated by the authors from the relevant units and field sites.

3. Thank you for stating the following financial disclosure:
   "No Funder, Fee will be paid by authors"

   At this time, please address the following queries:

   a) Please clarify the sources of funding (financial or material support) for your study. List the grants or organizations that supported your study, including funding received from your institution.
   b) State what role the funders took in the study. If the funders had no role in your study, please state: “The funders had no role in study design, data collection and analysis, decision to publish, or preparation of the manuscript.”
   c) If any authors received a salary from any of your funders, please state which authors and which funders.
   d) If you did not receive any funding for this study, please state: “The authors received no specific funding for this work.”

Response:

a) This study is supported partially by Joint **fund** key support project NSFC (u1911205) and the Beijing Research Institute of Chemical Engineering and Metallurgy through the project A60-3.

b) The funders help study design, data collection and analysis, decision to publish, preparation of the manuscript.

c) Yes, we have received a salary from “project NSFC (u1911205)” “the Beijing Research Institute of Chemical Engineering and Metallurgy through the project A60-3”.

d) I have received the funding for this study.

4. Please note that funding information should not appear in the Acknowledgments section or other areas of your manuscript. We will only publish funding information present in the Funding Statement section of the online submission form. Please remove any funding-related text from the manuscript.

Response: Thanks a lot. We have modified the Acknowledgement part as per suggestion.

Acknowledgement:

The authors would like to thank Mr. Yiwei Li and Prof. Jili Wen from Beijing Research Institute of Chemical Engineering and Metallurgy for refining this research work. Editors and two anonymous reviewers are also thanked for the valuable comments in improving the quality of the manuscript.

5. We note your Data Availability statement: "This research paper is part of research project (support project NSFC (u1911205) and the Beijing Research Institute of Chemical Engineering and Metallurgy through the project A60-3). Therefore, without permission of the funding agency and PI of the project data cannot be uploaded."

   PLOS journals require authors to make all data necessary to replicate their study’s findings publicly available without restriction at the time of publication. When specific legal or ethical restrictions prohibit public sharing of a data set, authors must indicate how others may obtain access to the data.

   Please address the following:
   a) Provide a point of contact, preferably an email, for Beijing Research Institute of Chemical Engineering and Metallurgy. Please note that this point of contact cannot be an author of this study.

Response: Thanks. There is an email of concerned persons form the Beijing Research Institute of Chemical Engineering and Metallurgy: 13932100832@163.com.

6. We note your response regarding our copyright query reads as follows: "Figure-1 is developed by the authors. Therefore, copyright section is not applied in this case."

   If Figure 1 was created and developed by the authors, we note it is still possible for this figure to have been developed using outside, potentially copyrighted, sources. As such, please make sure to respond to the below prompts. We will not be able to proceed until you respond:

   A. For the above figures, were any shapefiles or basemaps obtained? If so, where were they obtained? Please provide any relevant links as well as any relevant copyright information.

   B. For the above figures, were any external data utilized in the creation of these figures? If so, where were they obtained? Please inform us if the data was collected or produced by the authors. Please also provide any relevant links as well as any relevant copyright information.

   C. Was any software (e.g. ArcGIS) used in the creation of these figures?

Response: Figure 1 was redrawn by the author based on regional data. ArcGIS 10.8 was employed to develop the figure. The shape files were created by the authors using the base map as reference to draw administration boundaries. The figure is not taken from some paper or some monograph. Our work is a new work in the field, these drawings have not yet established a "link".

**Reviewer Comments**

**Comments Reviewer #1**

The authors have carried out a study on "Assessment of groundwater quality with reference to minerals in situ leaching uranium in Bayanwula mining area, China". However, after reviewing this manuscript, there are the following problems:

1.Abstract: Please refine the main research highlights to improve readability, otherwise it will reduce the quality of your manuscript. The current summary is too long.

RESPONSE： The authors are thankful to the reviewer for the helpful comments on improving our manuscript. we have revised the entire document to contextualize our findings and provide a more elaborate understanding of this research work and their implications as per the reviewer's suggestion. The entire document has been rearranged and rewritten.

We have revised the abstract. The main advantages of this study have been included in the abstract and introduction. ( line 12 to 32, line 130 to 152)

2. Introduction: It is necessary to reorganize the structure and sort out the current research status. The current introduction structure cannot effectively guide readers in reading the manuscript. I have annotated the details in the attachment.

RESPONSE： We have further summarized the current research status and reorganized the introduction as per the reviewer's suggestion. (line 40 to 152).

3. In section of 3.4 Initial parameters of the model, there are too many chapters and each paragraph has very little content. It is recommended to integrate them.

RESPONSE： We have updated the chapters and rearranged them as per the reviewer's suggestion. (Chapter’3.2 mathematical model).

4.What is the basis for the author's determination of minerals in the manuscript?

RESPONSE： Thanks for your comments. This section is included in this manuscript to give the actual site field understanding.

5.3.4.5 Model simulation accuracy and verification, There are many writing errors.

RESPONSE: We have updated the model accuracy and verification as per the reviewer's suggestion. (Line 412 to 434)

6.4. Results and discussions, it is just an explanation of the results, lacking in-depth discussion. In addition, the entire writing process is a bit chaotic and difficult to understand.

RESPONSE： Thanks a lot. We adjusted it according to two parts: fluid migration and mineral change. We add to the discussion of possible mineral transformation relationships as per the reviewer's suggestion.

7.Conclusions: 1.: Please refine the main research highlights to improve readability, otherwise it will reduce the quality of your manuscript.

RESPONSE：We rearranged the main research highlights of this research as per the reviewer's suggestion.(line 670 to 672)

In addition, there are two important issues:
(1)Suggest adding line numbers and page numbers, otherwise it will increase the difficulty of the review.

RESPONSE: The line numbers and page numbers has been added in revised manscript.
(2)There are too many writing errors and syntax error in the manuscript, and the manuscript language needs to be improved by professionals or institutions, otherwise it is not suitable for publication.

RESPONSE： Modified as per instructions of entire paper

**Comments Reviewer #2**

1.Introduction part is illogical. The reason for the research has not been clarified. What is the novelty in the manuscript? Methodology or study area?

RESPONSE： The authors are grateful for the reviewer’s critical comments regarding the manuscript’s improvement. Introduction part has been modified and rearranged as per reviewer’s guideline and comments. We addressed all your comments and modified our manuscript. We compare the innovation of this study with the gap of previous research in the introduction part.

2. Geological map and section are absent.

RESPONSE： The stratum structure of the study area is relatively flat; we showed the geological structure of the study area in a vertical sequence diagram in Figure 2 and Figure 3.

3. water-bearing rock should be replaced by aquifer.

RESPONSE： Thanks a lot. We replaced the “water-bearing rock” with “aquifer”.

4. Uranium orebody is not mentioned in Study area.

RESPONSE： The uranium orebody has been included and mentioned in the legend ”Sandstone uranium deposits” in Figure-1.

5. 40~60m is not correct writing in English.

RESPONSE： Modified in line 167 to 190 as per reviewer’s guideline and comments.

6. Groundwater flow direction is missing in Hydrogeological conditions.

RESPONSE： Thanks for your comments. The study area is in a flat high plain with week background groundwater flow. The main impact comes from manual extraction. We have included the groundwater flow direction in Figure 4.

7. The English should be polished. Too many very long sentences make the manuscript very hard to be understood.

RESPONSE： Modified as per instructions of entire paper

8. The results of numerical simulation are not robust. More evidence is needed to prove it.

RESPONSE： We have revised the results and included model accuracy and verification in line number 412 to 434 as per reviewer’s guideline and comments.
